# A transferable active-learning strategy for reactive molecular force fields†

**DOI:** 10.1039/d1sc01825f

**Published:** 2021-07-05

**Authors:** Tom A. Young, Tristan Johnston-Wood, Volker L. Deringer, Fernanda Duarte

**Affiliations:** Chemistry Research Laboratory, University of Oxford Mansfield Road Oxford OX1 3TA UK fernanda.duartegonzalez@chem.ox.ac.uk; Department of Chemistry, Inorganic Chemistry Laboratory, University of Oxford Oxford OX1 3QR UK volker.deringer@chem.ox.ac.uk

## Abstract

Predictive molecular simulations require fast, accurate and reactive interatomic potentials. Machine learning offers a promising approach to construct such potentials by fitting energies and forces to high-level quantum-mechanical data, but doing so typically requires considerable human intervention and data volume. Here we show that, by leveraging hierarchical and active learning, accurate Gaussian Approximation Potential (GAP) models can be developed for diverse chemical systems in an autonomous manner, requiring only hundreds to a few thousand energy and gradient evaluations on a reference potential-energy surface. The approach uses separate intra- and inter-molecular fits and employs a prospective error metric to assess the accuracy of the potentials. We demonstrate applications to a range of molecular systems with relevance to computational organic chemistry: ranging from bulk solvents, a solvated metal ion and a metallocage onwards to chemical reactivity, including a bifurcating Diels–Alder reaction in the gas phase and non-equilibrium dynamics (a model S_N_2 reaction) in explicit solvent. The method provides a route to routinely generating machine-learned force fields for reactive molecular systems.

## Abstract 2

An efficient strategy for training Gaussian Approximation Potential (GAP) models to study chemical reactions using hierarchical and active learning.

## Graphical abstract

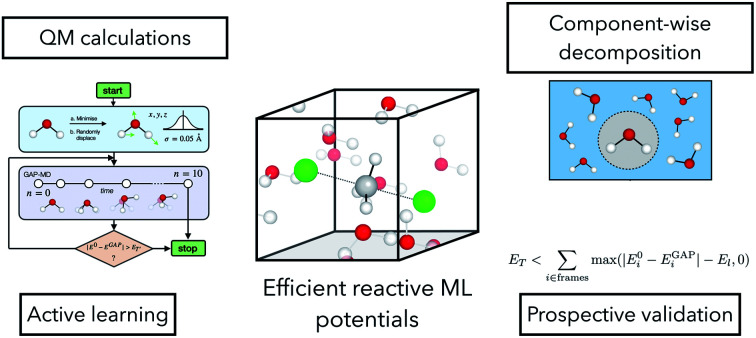

## Introduction

Molecular simulations are a cornerstone in computational chemistry, providing dynamical insights beyond experimental resolution.^1^ Realistic simulations of (bio)chemical reactions require the inclusion of the chemical environment where they occur (*e.g.* solvent and/or enzyme) and often extended timescales. Therefore, generation of accurate and efficient approaches has been central to the development of this field.

Empirical interatomic potentials (force fields), in combination with molecular dynamics (MD) or Monte Carlo (MC) simulations, have been widely used to sample the potential-energy surface (PES). However, they are limited in accuracy and transferability.^2^ Moreover, most of these potentials are parameterised for isolated entities with fixed connectivity and thus unable to describe bond breaking/forming processes. In contrast, *ab initio* methods provide an accurate description of the PES, which is particularly critical for reactions in solution. However, because of their high computational cost and unfavourable scaling behaviour, they are limited to a few hundred atoms and simulation times of picoseconds in *ab initio* molecular dynamics (AIMD) at the DFT level, and practically impossible at the computational ‘gold-standard’ [CCSD(T)].^3^

Machine learning (ML) approaches have the potential to revolutionise force-field based simulations, aiming to provide the best of both worlds,^4–6^ and have indeed begun to provide new insights into a range of challenging research problems.^7–16^ The development of an ML potential applicable to the whole periodic table mapping nuclear coordinates to total energies and forces is, however, precluded by the curse of dimensionality. Within small chemical subspaces, models can be achieved using neural networks (NNs),^6,17–21^ kernel-based methods such as the Gaussian Approximation Potential (GAP) framework^22,23^ or gradient-domain machine learning (GDML),^24^ and linear fitting with properly chosen basis functions,^25,26^ each with different data requirements and transferability.^27^ GAPs have been used to study a range of elemental,^28–30^ multicomponent inorganic,^31,32^ gas-phase organic molecular,^13,33^ and more recently condensed-phase systems, such as methane^34^ and phosphorus.^35^ These potentials, while accurate, have required considerable computational effort and human oversight. Indeed, condensed-phase NN^36,37^ and GAP fitting approaches typically require several thousand reference (“ground truth”) evaluations.

Active learning (AL), where new training data is added based on the current state of the potential, has been used for generating databases and accelerating the fitting process.^31,38–42^ Notable examples in materials modelling include an early demonstration of a “query-by-committee” approach in fitting a high-dimensional NN potential for elemental copper,^39^ the fitting of Moment Tensor Potential^26^ models^43^ to predict elemental crystal structures^38^ and multicomponent alloys,^40^ and the deep potential generator (DP-GEN)^44,45^ that provides an interface to deep NN potential models for materials.^46^ AL schemes have also been combined with GP based force fields including GAP,^47^ and included within a first-principles MD implementation such that it allows the “on the fly” fitting of force fields for a specific simulation system.^48,49^

Efficient approaches to generate reactive ML potentials become even more important when exploring chemical reactions in molecular systems, which often require a description at a computational level beyond DFT, and therefore require reference data at the same level. Very recently, AL approaches have started to be adopted for fitting reactive potentials for organic molecules based on single point evaluations at quantum-chemical levels of theory. Notable examples include the modelling of gas-phase pericyclic reactions,^12^ the exploration of reactivity during methane combustion,^50^ and the decomposition of urea in water.^41^

In the present work – with a view to developing potentials to simulate solution phase reactions – we consider bulk water as a test case and develop a strategy which requires just hundreds of *total* ground truth evaluations and no *a priori* knowledge of the system, apart from the molecular composition. We show how this methodology is directly transferable to different chemical systems in the gas phase as well as in implicit and explicit solvent, focusing on the applicability to a range of scenarios that are relevant in computational chemistry.

## Results and discussion

Despite GAP fitting being increasingly used for inorganic systems, we found that the same fitting strategies did not easily transfer to the description of complex molecular environments. Even with a high correlation and low error on energies in unseen test data, some potentials were not stable for more than a few femtoseconds. In the following section, we therefore outline a training strategy along with a prospective error metric to develop robust models for gas-phase and condensed-phase molecular systems.

### A prospective error metric

The initial step in validating supervised machine learning (ML) tends to follow the splitting of a dataset into training and test sets, training the model, then evaluating its performance on the test set with a squared error (RMSE/MSE) or a correlation (*R*^2^) metric. As with model overfitting, this ‘retrospective’ validation strategy ultimately limits the applicability of these models.^51–57^ In an ML potential, the minimum required domain of applicability is the region of configuration space likely to be sampled during a simulation with the potential. However, this region is not known *a priori*, making the choice of test data problematic if not impossible for use in a standard train/test data split approach. In addition, one would also like to ensure high accuracy in regions sampled on the ground truth surface (especially for early versions of an evolving potential), but being able to quantify this accuracy requires dynamics at the ground truth method level in the first place, which is much more expensive than sampling with an efficient potential.

Using a train/test set split with high structural similarity between the two sets can lead to highly misleadingly accuracy whenever the potential is to be taken outside the training region in computational practice. For example, splitting an AIMD trajectory of water into a training and test set with an odd/even frame split (50 : 50) and training a simple GAP model yields an energy error on the order of 1 kcal mol^−1^ (Fig. S1a†). However, simulations with this potential in the same configuration space sample unphysical configurations within 10 fs (Fig. S1b†), making an RMSE over *a priori* test data an insufficient metric in quantifying the quality of a potential.

Considering that single-point reference energy evaluations are reasonably cheap, a ‘prospective’ validation scheme is possible, where the error metric operates in the configuration space sampled in a simulation. With this in mind, we propose a temporal cumulative error metric (*τ*_acc_, eqn (1)), defined as the time required for the cumulative error (absolute difference between true (*E*^0^) and predicted (*E*^GAP^)) to exceed a given threshold (*E*_T_); the larger *τ*_acc_, the more robust the potential. Note that the time for which a potential is stable in MD can far exceed *τ*_acc_, as shown in the following. Here only errors above a lower-bound threshold value (*E*_l_) contribute to the cumulative error. The lower threshold is required to account for the residual error that is due to the finite radial cut-off of the model. In the following we take *E*_T_ to be 10 times *E*_l_, but it may be adjusted depending on the simulation context.1
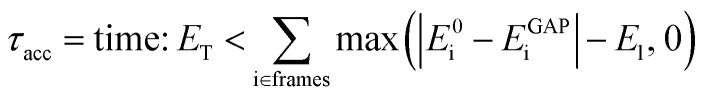


**Fig. 1:**
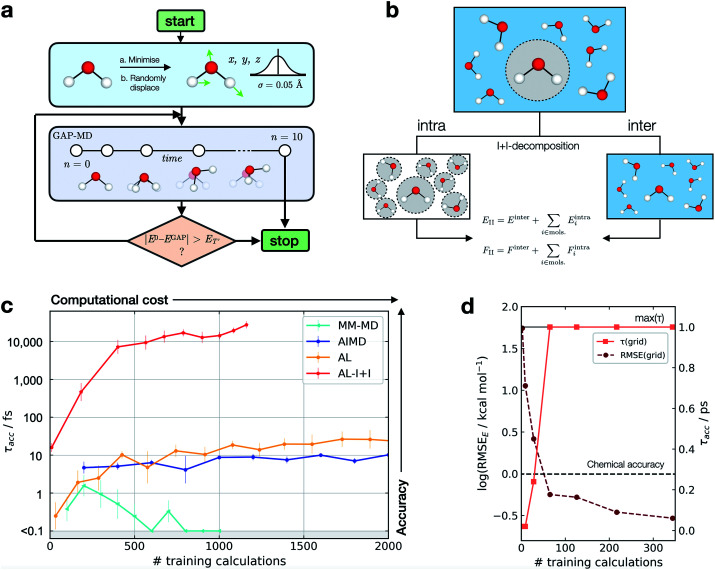
Active learning of machine-learning potentials for liquid water. (a) Schematic of the active learning loop implemented for fitting GAP models, where the GAP-MD exploration is run for *n*^3^ + 2 femtoseconds, where *n* (the number of evaluations) is incremented after each time the error is evaluated. (b) Schematic illustrating the separation into inter- and intra-molecular terms (I + I) for a bulk water system; these are described by separate GAP models (here, using the same method to obtain the reference data), and then added to give the combined prediction for energies, *E*, and forces, *F*. (c) Learning curves for a bulk water GAP model using different training strategies. *τ*_acc_ with *E*_l_ = 0.1 eV, *E*_T_ = 1 eV, 10 fs interval, 300 K, from the same random minimised configuration of 10 waters in a 7 Å cubic box. Error bars quoted as the standard errors in the mean from 5 independent repeats. The horizontal axis denotes the number of evaluations in training data generation. See Tables S1 and S2† for detailed methods. DFTB(3ob) ground truth. Minimum *τ*_acc_ is shown as 0.1 fs to enable plotting on a log scale. (d) Water monomer model training performance as characterised by *τ*_acc_ and RMSE over the full 3D PES; see Fig. S2† for details.

This metric has several advantages in that (a) it ensures that a potential with high accuracy will result in stable dynamics; (b) it allows the user to specify the level of accepted error according to the quality of the training method, thus not penalising where the error is within the difference between the ground truth and the true PES (*i.e.* a larger threshold may be suitable for a less accurate reference method); (c) it penalises large errors, even if they only occur for single configurations, which is important as such errors may lead to instabilities in the ML-driven MD trajectory and (d) it enables a quoted accuracy to include regions that may not be accessible to direct evaluation at the ground-truth level (*e.g.* long-time behaviour). Overall, this metric depends on the lower bound and total error, interval between evaluations, and the simulation on which it is evaluated; so while not unique, it is – crucially – prospective. We found this metric to be essential in developing an efficient training strategy and accurate potentials for bulk water (Fig. 1).

### Water models

For bespoke ML potentials to be routinely developed for molecular systems, one would hope to complete the data generation, model training, and know the accuracy of the resulting potential within a matter of hours to days. With this in mind, here we train GAP models to simulate bulk water, aiming to minimise the number of required ground truth evaluations as well as the required human intervention, while maximising stability (measured by *τ*_acc_). A selection of training strategies is discussed in the following paragraphs and their results are outlined in Fig. 1.

We initially employed training strategies found to work well in elemental materials by, for example, fitting a combined potential with two- and three-body GAPs. However, this approach was found to be detrimental to the potential's stability. This can be understood considering a water dimer (HO–H_c_⋯OH_2_); here, a two-body description that treats the two O–H_c_ interactions on the same footing is a poor approximation, in view of the different order of magnitude between the interactions at their respective minima (Fig. S3†). Therefore, we decided to proceed employing a smooth overlap of atomic positions^58^ (SOAP) descriptor for an exclusively many-body description of atomic environments (with the exception of AL-I + I, which uses 2 + 3 body for the intramolecular component as discussed later).

We also explored different approaches to generate the database and their influence on the generated potential. An emerging approach to generate training data for elemental GAPs is to initialise the database with randomised configurations (with reasonable constraints, as in *ab initio* random structure searching^59^), and to gradually explore configuration space with evolving versions of the potential (see, *e.g.*, ref. 60). However, randomly placing water molecules does not in itself afford a stable potential. A similar result is observed when the most diverse configurations are selected using the CUR algorithm^60,61^ (Fig. S4†) or when applying intramolecular displacements, following minimisation (Fig. S4†). Selecting frames from classical MD simulations at temperatures of 100–1000 K was also found to be an ineffective strategy (Fig. S5†), reaching *τ*_acc_ of only a few fs (“MM-MD”, Fig. 1c). This is in line with the results reported in ref. 13. Note that this is not because the GAP cannot fit reference energies and forces from MM configurations (Fig. S6†), but because of a poor configuration space overlap with the ground truth PES (Fig. S7†). Selecting configurations from an AIMD simulation at 300 K (AIMD, Fig. 1c) was an improvement over training on random and MM-generated configurations, with *τ*_acc_ ∼ 10 fs. However, by adding additional AIMD configurations the increase in accuracy saturates quickly even if those are obtained at higher temperatures (Fig. S8†). Using AIMD configurations can also involve a significant cost (requiring thousands of evaluations). Finally, active learning from only a few randomly generated configurations provides a modest uplift in accuracy (AL, Fig. 1c), with accuracy on-par with GAP trained on AIMD configurations at a third of the required reference data.

Only when the relevant length and energy scales of the system are decomposed by treating intra- and inter-molecular components separately (Fig. 1b) a potential that is stable for picoseconds is obtained (AL-I + I, Fig. 1c). We note that this approach is related to the hierarchical fitting of GAPs^34,63^ and related ML models^33,64–66^ using different levels of computational approaches, and the decomposition strategy by Wengert and co-workers.^67^ In the present work, we employ the same ground-truth method throughout rather than combining different levels of theory for the input data, but as in prior studies we describe the stronger (*e.g.*, covalent) and weaker intermolecular terms with separate fits that are afterwards combined to give the final model. The intramolecular GAP for water contains only 2- and 3-body terms and the training data are chosen using an evenly spaced grid over the full 3-atom PES (8 × 8 × 8 grid points in *r*_OH_ and *r*_HH_, ∼0.1 Å spacing, Fig. S9†). Energy and force evaluations of this potential are a simple sum of intra- and inter-molecular terms, but require the former to be evaluated in an expanded simulation box to ensure no non-bonded hydrogen atoms are present within the cut-off radius of the 2- and 3-body descriptors on oxygen (Fig. S10†). Here the intramolecular PES is fairly low-dimensional, so a full and reasonably dense grid is available, which in turn allows us to define an error measure over the whole PES, where we find the error to be inversely correlated with *τ*_acc_ (Fig. 1d). Using an acceptable error of 0.2 kcal mol^−1^ per H_2_O molecule for a description of bulk water, which is similar to that achieved in a recent NN fit of water,^36^ we find that this potential affords *τ*_acc_ > 10 ps with just a few hundred ground truth evaluations (AL-I + I, Fig. 1c). To put this value in context, we measured *τ*_acc_ for the fully reactive water NN of Cheng *et al.*,^36^ which was trained on ∼7000 reference configurations (DFTB energy/forces) and has shown to provide a highly accurate water model over multiple states. For this state-of-the-art ML potential, a *τ*_acc_ value of 7.6 ± 0.7 ps for liquid at 300 K is obtained, comparable to the one obtained for the new AL-based potentials of the present work (>10 ps). Of course, direct comparison requires caution because the two potentials are different in scope: the NN potential employs a large reference database to develop a general water model, whereas the present study targets robust potentials for liquid water with minimal computational effort, in turn allowing the user to apply similar approaches to other chemical systems (as will be shown below).

**Fig. 2:**
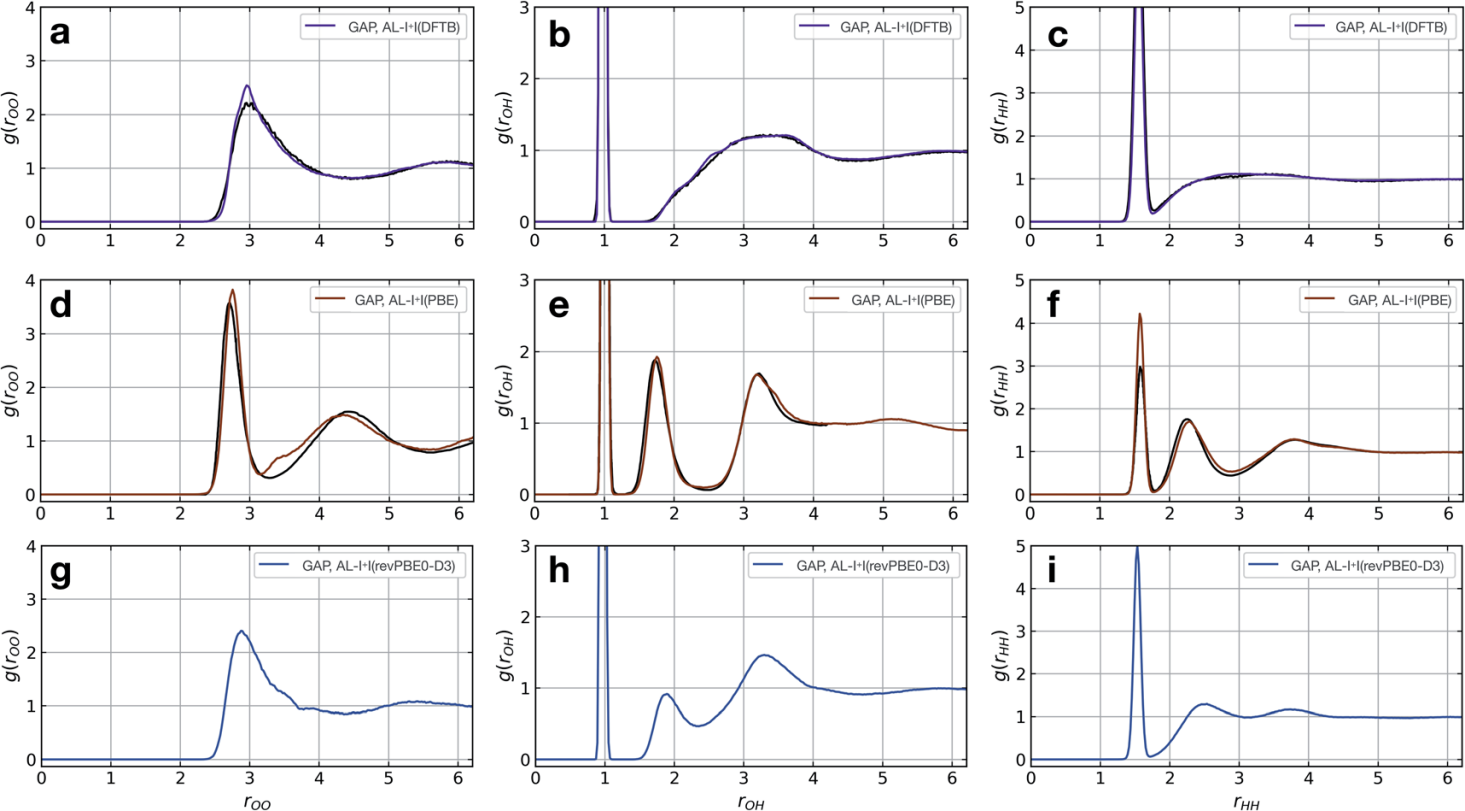
Liquid water simulations. Active learning of bulk water models at various levels of theory. Shown here are O–O, O–H, and H–H RDFs from NVT MD simulations of 64 water molecules in a 12.42 Å cubic box, with ground truth (black) and GAP (purple/red) simulations. (a–c) DFTB(3ob params) ground truth, 100 ps, 300 K, *r*^SOAP^_c_(O) = 3 Å. (d–f) DFT(PBE) reference RDF data extracted from ref. 62, 30 ps, 330 K, *r*^SOAP^_c_(O) = 3.5 Å. (g–i) DFT(revPBE0-D3) GAP, 30 ps, 330 K, *r*^SOAP^_c_(O) = 4.0 Å.

The model fitted using our approach (AL-I + I) yields radial distribution functions (RDFs) in good agreement with the ground-truth method, initially chosen to be DFTB, both considering the location and intensities of the peaks corresponding to the first and second coordination shells (Fig. 2a–c). This is despite the relatively short-range atomic cut-offs (3 Å, O only) used. Only in the O–O pair RDF there is a slight deviation from the DFTB ground truth, precisely where the potential is zero outside the 3 Å cut-off radius of the SOAP descriptor. Interestingly, for a DFT-quality GAP simply re-evaluating energies and forces on DFTB-derived active-learnt configurations is insufficient, with the DFTB configurations being high in energy at the DFT level (∼5 eV, Fig. S11†). However, applying an active learning strategy with a PBE reference method and a slightly larger 3.5 Å cut-off generates excellent agreement with the AIMD simulation from ref. 62, in only a few hours of total training time (Fig. 2d–f). At this level of theory, the local structure of liquid water is predicted largely correctly, with two distinct peaks in the O–H RDF, corresponding to first and second solvation shells with the largest deviation from the ground truth again in the O–O pair around the descriptor cut-off. The real significance, of course, is in moving to more accurate ground-truth methods, for which a full MD simulation would not be straightforward: indeed, using the same method, a hybrid DFT-quality water model can be generated within a few days, which would be inaccessible with other methods (the generation of the GAP model required ∼5 days on 20 CPU cores, Fig. 2g–i). These results suggest that the training strategy (and hyperparameter selection) presented here is suitable independent of the reference method.

### Other solvent systems

Organic reactions often take place in solvents other than water. Using an identical training strategy to the one described for water, we trained GAPs for a selection of organic solvents with various types of intermolecular interactions. To quickly generate the reference simulation data for this proof-of-concept, a DFTB ground truth is employed; chlorinated solvents were not selected due to a large discrepancy between the DFTB-generated and experimental C–Cl bond dissociation energy (Fig. S12†). A uniform grid over the intramolecular PES is now no longer possible; thus, AL is used to develop an initial intramolecular potential trained using GAP-MD at 1600 K (Fig. S13†). This temperature is used to sample higher-energy configurations more efficiently. In all cases, only hundreds of ground truth evaluations were necessary to generate GAPs affording stable dynamics, with *τ*_acc_ values on the order of picoseconds (Table 1, Fig. S14, ESI Section S1†). For a representative example, the computed RDFs for acetonitrile compare well with the ground truth (Fig. S15†). As with the water models above, to quantitatively evaluate bulk properties training an accurate reference method and the inclusion of nuclear quantum effects would be necessary.^34,36^ Nevertheless, this example demonstrates that the training method is applicable to a range of chemical systems beyond water. In this sense, the strategy presented here can be considered “transferable” as it can be directly applied to obtain ML potentials for other chemical systems. This should not be confused with general ML potentials which are aimed to describe different systems.

**Table 1:** Average number (*N*) of *total* ground truth evaluations (over 5 repeats quoted with a standard error in the mean) required to obtain a potential with *τ*_acc_ > 3 ps, where *E*_T_ = 1 eV, *E*_l_ = 0.1 eV, 300 K. All SOAP descriptors used 3.0 Å cut-offs; they are centred on the stated atomic species, and include all atoms within the neighbourhood of those atoms (including hydrogen). See Table S3 for more detailed parameters

Solvent	SOAP descriptors centred on	*N*_intra_	*N*_inter_
Acetonitrile	C, N	269 ± 12	120 ± 60
Methanol	C, O	221 ± 13	292 ± 49
Acetone	C, O	566 ± 80	359 ± 29
Pyridine	C, N	249 ± 36	243 ± 11
Ammonia	N	38 ± 40	109 ± 24

### Aqueous Zn(ii)

**Fig. 3:**
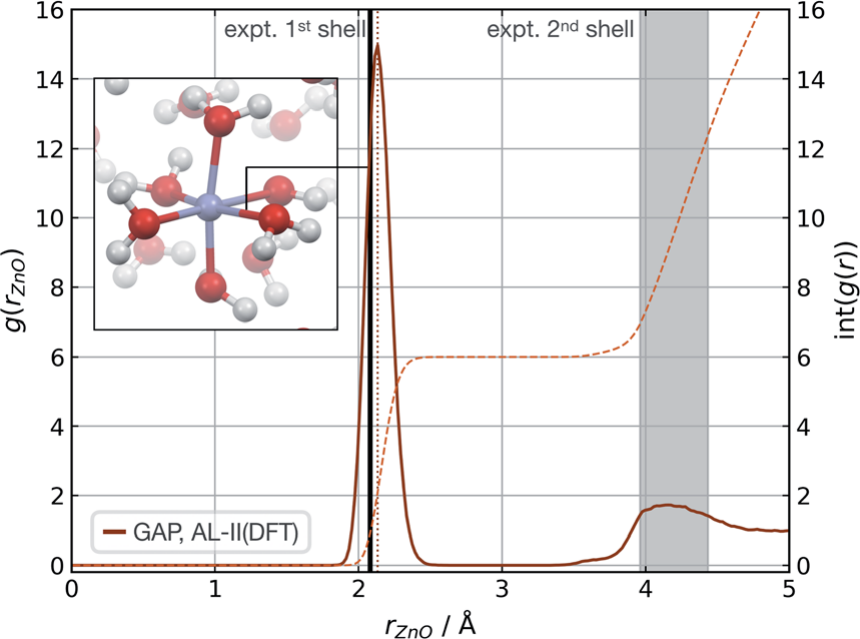
Zn_(aq)_ simulation. Zn–O radial distribution function averaged from 1 ns of cumulative (10 × 100 ps) NVT MD simulations of Zn(ii) in aqueous solution at 300 K, with the experimental modal Zn–O distance shown in black. Experimental (X-ray diffraction) Zn–O distances from ref. 69, octahedral first hydration shell. The shaded area denotes the range of experimental second hydration shell (ref. 69 and cited within). GAP trained as those in Table 1 using a PBE/400 eV ground truth, intra-Zn(H_2_O)_6_ used a O-centred SOAP *r*_c_ = 3.0 Å and inter *r*_c_ = 4.0 Å.

Modelling metal ions in solution remains one of the main challenges for general-purpose force fields.^68^ Historically, metal ions have been described by fitting van der Waals parameters to reproduce RDFs and hydration free energies of aquo complexes, which are expected to be transferrable to more chemically complex environments. However, while simple, these models have often led to unstable simulations or poorly describe structural properties.^68^ Considering these challenges and their relevance in biomolecular modelling, we decided to use our strategy to generate a GAP for aqueous Zn(ii) ion as a representative system. Here the system was decomposed into a [Zn(H_2_O)_6_]^2+^ cluster and the remaining water molecules. A strategy identical to the one described for water was used, training the intermolecular interactions separately with a 4.0 Å intermolecular cut-off for the oxygen atoms. Using this potential, MD simulations were propagated at 300 K reproducing the experimental^69^ coordination number (CN = 6), and Zn–O distances of both the first (2.08 Å) and second hydration shells without further optimisation (Fig. 3).

The accuracy of the local structure compared to experiment in the first and second solvation shell indicates that this partitioning is effective at capturing both strong dative M–OH_2_ interactions and weaker hydrogen bonding effects. From random points in the configuration space of [Zn(H_2_O)_6_]^2+^ and 20 water molecules (intermolecular distances >1.7 Å, 10 Å cubic box), *τ*_acc_ reached 0.5 ps (*E*_l_ = 0.8 kcal mol^−1^ per H_2_O, 20 fs interval). Note this value is far short of the 100 ps simulations performed to generate the RDF and illustrates that a potential may be ‘stable’ and not sample any high energy regions for *t* ≫ *τ*_acc_. Here, the potential for the Zn–water cluster was trained on almost 1000 configurations, suggesting that tens of atoms per component may be the upper limit in dimensionality for which a model can be trained within a day.

### Metallocage dynamics

With a method capable of generating high-quality potentials for modestly sized chemical systems, we next demonstrate the applicability of the strategy to investigate a supramolecular metallocage consisting of >100 atoms including metal ions. As a representative example, we selected the [Pd_2_L_4_]^4+^ metallocage architecture (L = organic pyridine-based ligand), which occupies a prominent place in supramolecular chemistry. Previously, we have studied this system due to its catalytic proficiency in Diels–Alder reactions employing both classical molecular dynamics and DFT modelling.^70^ The different flexibility of two similar cage architectures was found to be key in explaining their contrasting catalytic activity.

**Fig. 4:**
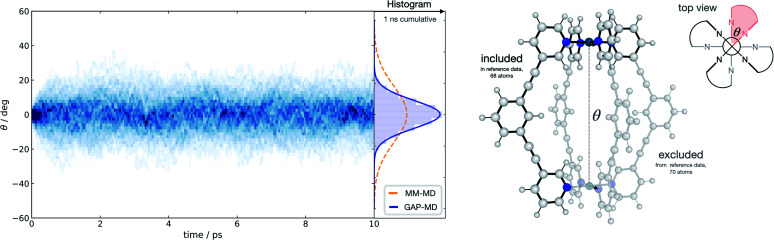
Metallocage dynamics. Temporal twist angle *θ* for an [Pd_2_L_4_]^2+^ metallocage (right) obtained from 100 independent GAP-MD trajectories (each run for 10 ps at 300 K), GAP trained on a 68-atom [PdL(py)_3_]^2+^ system (py = pyridine), representative of the whole metallocage as shown, at 500 K with a 0.2 eV error threshold for the active-learning protocol. Time-dependent histogram generated from 50 fs time chunks over the whole 10 ps time period. PBE0-D3BJ/def2-SV(P) ground truth surface.

Taking advantage of the symmetry in the system, a representative fragment containing one full ligand and three pyridine molecules coordinated to a Pd^2+^ metal ion (68 atoms) was used to fit a GAP for the entire cage (138 atoms) in the gas phase. This potential was trained in a few days (∼1400 CPUh). We used the resulting GAP to perform nanosecond MD simulations on the whole metallocage at 300 K in the gas phase. This simulation took one day and ∼100 CPUh to complete. For comparison, an equivalent AIMD simulation would take around 50 years with the reference level of theory employed here. The flexibility of the system was monitored and compared to the one obtained using classical MD simulations in dichloromethane solvent.^70^ Compared to classical MD simulations, using helicity as a measure of flexibility, our potential describes the cage as being more rigid; this suggests that the classical potential overestimates the dynamic flexibility (Fig. 4). This difference is expected as the classical potential has no C–C≡C–C dihedral barrier, which is presumably correctly captured in the GAP. This example illustrates the general applicability of the approach to increasingly complex systems, where the training of a simpler but representative fragment is sufficient to capture the relevant features of the full system.

### Reaction dynamics in gas and solvent phase

The high dimensionality and ensuing flexibility of ML potentials make them highly suitable to study reaction dynamics – the latter usually require many costly electronic structure calculations to obtain atomic-level descriptions of reaction mechanisms, solvent effects or post-transition state (TS) dynamics.^71,72^ In the following section, we show that our data-efficient strategy enables accurate reactive potentials (*τ*_acc_ > 100 fs) with only a few hundreds of DFT evaluations for a set of prototypical organic reactions.

#### Gas phase bimolecular nucleophilic substitution

The S_N_2 nucleophilic substitution reaction is fundamental in organic chemistry and has been extensively studied using AIMD and analytically fit PES.^71,73^ However, even with efficient approaches to fitting PES, AIMD methods still require tens of thousands of energy evaluations.^74^ Here, we generated a reactive GAP to study the reaction between chloride and methyl chloride as a prototypical case often employed to validate QM/MM reactions and for which extensive literature exists (see ref. 75 and references therein). By initialising active learning from the transition state (TS), the true intrinsic reaction coordinate is reproduced to within 1 kcal mol^−1^ (Fig. S16†).

**Fig. 5:**
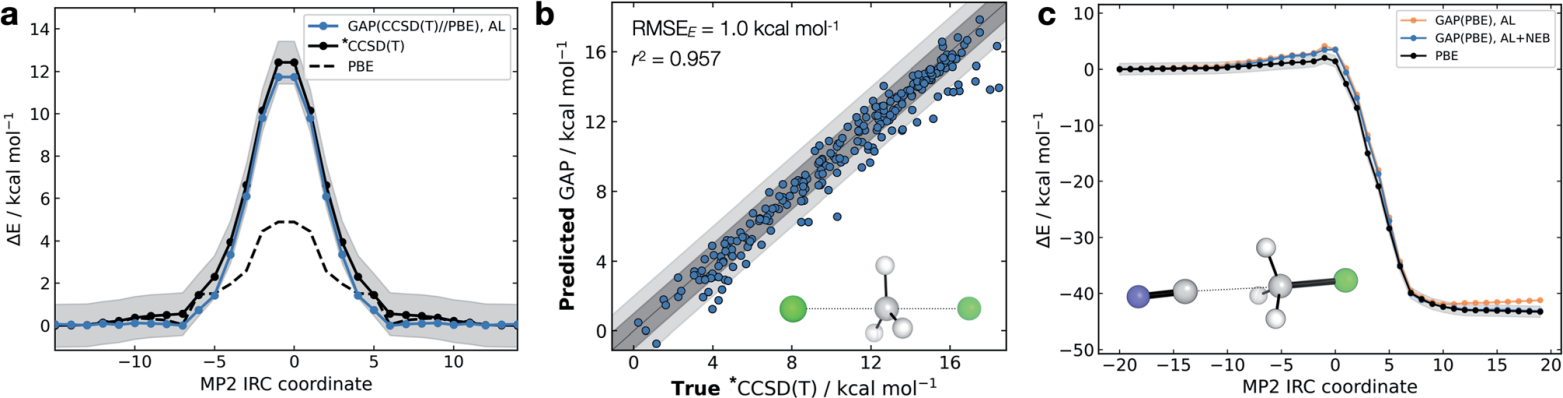
Gas-phase S_N_2 reactive dynamics. Energetics of a mode Cl^−^ + CH_3_Cl → Cl^−^ + CH_3_Cl S_N_2 reaction in the gas phase. (a) Predictions (GAP) and ground-truth (*CCSD(T) ≡ DLPNO-CCSD(T)/ma-def2-TZVPP) energy values on the MP2/ma-def2-TZVPP intrinsic reaction coordinate (IRC). Shaded region bounds the ‘chemically accurate’ (±1 kcal mol^−1^) region. (b) Parity plots of between GAP predictions and true energies from ten 100 fs GAP-MD trajectories initialised from the TS (300 K). Dark and light grey area bound the ±1 kcal mol^−1^ and ±2 kcal mol^−1^ error regions, respectively. (c) IRC for CN^−^ + CH_3_Cl → Cl^−^ + CH_3_CN but trained using uphill active learning, with nudged elastic band refinement; see Fig. S16† for additional details.

Interestingly, and unlike our previous attempt to generate a DFT-quality GAP by evaluating energies and forces on DFTB active-learnt configurations, here, uplifting a DFT-level GAP to an accurate wavefunction-level GAP is possible. This method allows coupled cluster-quality energy profile (Fig. 5a) and dynamics to be propagated from the TS with just 55 energy and (numerical) force evaluations at the CCSD(T) level (Fig. 5b and S16†). The resultant GAP is considerably more accurate than the underlying DFT energy profile (dashed, Fig. 5a). Active learning can also be initialised from an association complex and the IRC learned without prior knowledge of the TS. For the exothermic reaction between cyanide and methyl chloride, training from reactants and initialising velocities such that 11 kcal mol^−1^ (0.5 eV) was present in the breaking bond, the reaction is sampled in the training (Fig. S16c†). Relaxing a nudged elastic band (NEB) using the trained GAP over an interpolated path between reactants and products affords an IRC within chemical accuracy of the true profile (RMSE = 0.9 kcal mol^−1^, Fig. 5c). In this case, adding a NEB refinement step to the training is essential to adequately sample the product region and reach chemical accuracy in the energy of the product, and therefore in the predicted reaction energy (orange *vs.* blue lines Fig. 5c). Here, uplifting the GAP to CCSD(T) affords chemical accuracy in the minima and TS regions, although with a more limited accuracy in the region in between (Fig. S16d†); this is likely due to the large differences between the PBE and CCSD(T) surface in that region. Despite this, the uplifted profile is again considerably more accurate than the underlying DFT (Fig. S16d†).

#### Post-TS bifurcating pathway in a Diels–Alder reaction

**Fig. 6:**
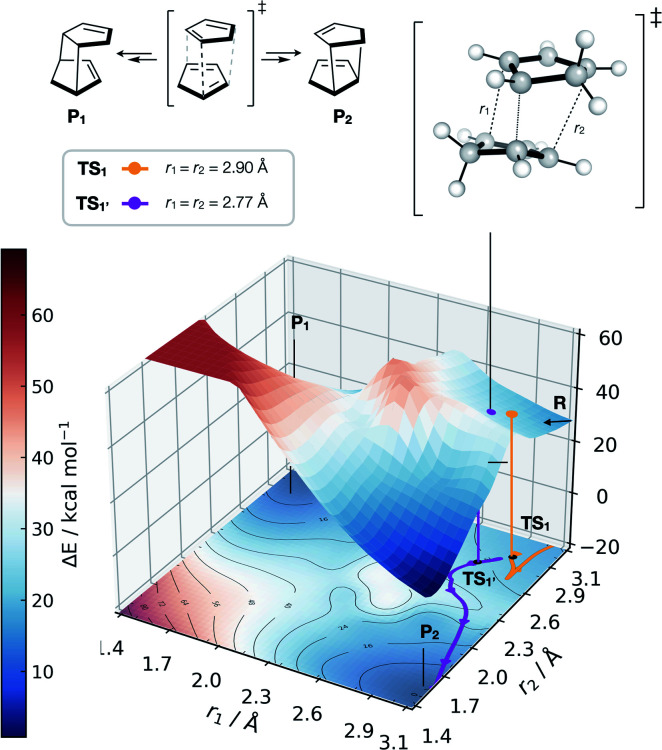
GAP dynamics on a bifurcating surface. 2D PES (B3LYP/def2-SVP) along the forming bond distances (*r*_1_, *r*_2_) in the dimerisation of cyclopentadiene. An example of GAP-propagated reactive dynamics (300 K) is shown from TS_1_ (7N in ref. 77), which leads to reactants (representative trajectory in orange), and from TS_1′_ which leads to products (a representative trajectory is shown in purple). 3D projection is truncated at 2.5 eV above the minimum for plotting. Interpolated surface used a cubic spline using scipy.interp2d with the raw surfaces shown in Fig. S21.† All trajectories shown in Fig. S18 and S20.†

GAPs for more complex reactions involving reactions that proceed on a bifurcating PES can also be trained. These reactions typically require AIMD simulations, where selectivity is determined from the average behaviour of many trajectories leading to either product. Other approaches have also been developed.^76^ We explored the dimerisation of cyclopentadiene, for which *endo* selectivity has been rationalised on the basis of bifurcating reaction pathways.^77^ Once again, initiating active learning from the literature TS (TS_1_, Fig. 6) and using a DFT method analogous to the one used in the original work by Caramella and co-workers we obtain a reactive potential from which 500 fs trajectories were propagated. Interestingly, we found that propagating the system from this TS did not afford any products (P_1_ or P_2_, Fig. 6), with all trajectories leading to the reactant state (Fig. S18†).

Further investigation and generation of the relaxed 2D potential energy surface over the two possible forming C–C bonds (*r*_1_, *r*_2_) leading to products provided a rather different surface to the one suggested in ref. 77, with a flat portion then an incline as *r*_1_, *r*_2_ shorten below 2.9 Å, with a steeply exergonic reverse reaction (intrinsic reaction coordinate, IRC, shown in Fig. S19†). As noted by Caramella, following the IRC forwards from TS_1_ the reaction proceeds to another TS_1′_ which is similar in energy (Δ*E* = 2 kcal mol^−1^). By training a GAP at 500 K and propagating GAP-MD from TS_1′_ and sampling the area of the PES around a valley-ridge inflection point (VRI), trajectories lead to the expected two products (*e.g.*, purple line, Fig. 6 and S20†). This example demonstrates that with no *a priori* knowledge, apart from the structure of TS_1_, the topology of the bifurcating surface can be revealed efficiently using GAP dynamics. This strategy is completely automated, requiring training of a few hours to days, thus providing a promising approach to routinely examine reaction dynamics in organic molecules.

#### Solution phase bimolecular nucleophilic substitution

**Fig. 7:**
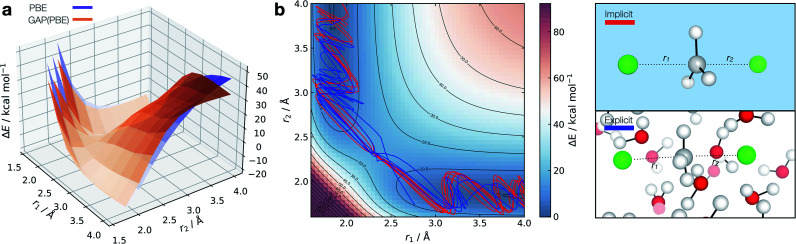
Solution-phase S_N_2 reactive dynamics. (a) True (CPCM(Water)-PBE/def2-SVP, purple) and GAP predicted (orange) relaxed 2D PESs in implicit solvent, zeroed to the transition state energy. (b) Reactive GAP MD trajectories (lines) propagated from the TS, trained on implicitly solvated configurations (CPCM(Water)-PBE/def2-SVP, red) and explicitly solvated configurations (PBE/400 eV, blue). Ground truth implicitly solvated surface (cubic interpolated, 5 : 1) underneath, with the error to the GAP prediction in Fig. S23.† Intramolecular GAP used C-centred (*r*^SOAP^_c_ = 6 Å) descriptor with the intermolecular explicit solvent using a SOAP on O (*r*^SOAP^_c_ = 3.5 Å) and Cl (*r*^SOAP^_c_ = 4.5 Å, Fig. S24†).

The ability to accurately describe bond-breaking/forming paths in the condensed-phase is crucial if this strategy is to be applied to increasingly complex processes, such as enzymatic reactions. Towards this goal, and having generated potentials for condensed-phase molecular systems and gas-phase reactions, we decided to extend our active learning strategy to explicitly solvated reactions; once again, using the S_N_2 reaction between chloride and methyl chloride as a test case. S_N_2 reactions have been used as a test case for a recent ML potential, but those studies have been limited to the gas phase and used thousands of training points.^21^ Literature examples of ML potentials to study reactions in explicit solvent are limited. Recently, Parrinello and co-workers reported an NN potential to study urea decomposition in water.^41^ Training a model for the implicitly solvated reaction proceeds in a similar way as for the gas phase analogue and affords a surface close to the ground truth (Fig. 7a).

Adopting an identical strategy to the one employed for condensed phase systems, the intra- and inter-molecular PES dynamics can be propagated from the TS and the effect of explicit solvation interrogated (Fig. 7b). This only requires knowing *a priori* the gas-phase TS for the training to be complete in explicit water. Interestingly, the behaviour in explicit water (blue, Fig. 7b) differs from the implicit counterpart (red, Fig. 7b). This can be understood considering that in implicit solvent reorganisation is instantaneous, which results in oscillations in the C–Cl bond characteristic of a gas phase reaction. In contrast, the dynamics are more complex in explicit solvent, with a slower transition from the product channel. Additionally, one of the 10 trajectories re-crosses the barrier after 170 fs of simulation (Fig. S22†), where the solvent has not reorganised to accommodate the anionic chloride yet, making the path to products shallower in energy.

The component-wise separation of the system also leads to the possibility of training to a more accurate *ab initio* surface for the gas phase reaction, in a similar way to QM/MM, but here a ML(A)/ML(B) partition is available where A and B are two different ground truth methods.^33^ Application of this kind of hierarchical ML potential fitting will be the subject of further work.

### Limitations

In its current implementation, this method is well suited for studying systems of up to 50 atoms if training is expected to be completed in a day, using a single node of 20 CPUs. For larger systems, the speed will depend on the complexity of the PES, where even using inexpensive methods thousands of training configurations and several iterations cycles may be needed to learn the different atomic environments. In contrast, training can be achieved efficiently for large systems with higher symmetry, and effectively lower dimensional PES. This is the case, for example, for the supramolecular cage shown above, which contains 138 atoms and can be modelled appropriately with a GAP that has been trained using a smaller system of only 68 atoms (Fig. 4). Furthermore, the current intra + intermolecular decomposition remains fixed throughout the simulation making the water potential generated initially incapable of auto-ionisation. Re-determining the connectivity (and therefore re-assigning the “intra” components) every few steps during the simulation might help to address this limitation. However, provided the model has been trained in a region where a chemical change has occurred, the molecular components in that region do not need to retain their connectivity. This is the case, for example, in the S_N_2 reaction shown in Fig. 7, where the connectivity changes between the two molecular units that constitute the solute.

## Conclusions

Studying dynamic processes and the effect of explicit solvation on chemical reactions demands a rapid method to develop bespoke force-field models with high accuracy. Here, we demonstrated that within the Gaussian Approximation Potential (GAP) machine learning framework, accurate and robust models can be developed efficiently for gas-phase and condensed-phase molecular reactions. Our strategy starts from a small number of randomly selected points in the configuration space, from which active learning training of intra- and inter-molecular components of the energy and forces is carried out. The developed method is publicly available (https://github.com/duartegroup/gap-train). We also define a prospective error metric, which is found to be crucial in developing robust active-learning-based potentials, whereas correlation on a predefined test set is insufficient to assess the quality of such a potential. We illustrated the generality of this approach by modelling bulk water, Zn(ii) in aqueous solution, and chemical reactions in the gas phase and explicit solvent, including post-TS cyclisation and S_N_2 reactions. The diversity of the examples presented here demonstrates the general applicability of the strategy and encourages applying this approach in the modelling of more complex reactions in homogeneous and heterogeneous environments.

## Methods

**Fig. 8:**
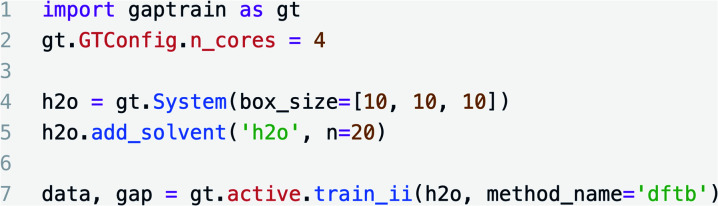
Example Python input script required to train a bulk water model from scratch at the DFTB level using four CPU cores.

All Gaussian Approximation Potentials (GAPs) were trained using the GAP and QUIP codes (singularity distribution, commit #66c553f) and a Smooth Overlap of Atomic Positions (SOAP)^58^ kernel with radial cut-off values defined in figure captions all with a smoothness (*σ*_atom_) of 0.5 Å; other hyperparameters defined in Table S1.† For a single component condensed-phase system such as water, two GAPs were fitted for the intra and intermolecular components, respectively, while for the solute–solvent systems, such as the S_N_2 reaction between chloride and methyl chloride, three GAPs were fitted: one for the gas-phase solute, a second for gas-phase solvent and a third one for the intermolecular interactions; see Table S2,† entry 6 for details. An example of the input script required to train a bulk water model is shown in Fig. 8. In all systems the intramolecular water potential was trained at the reference level on an evenly spaced grid (512 points, *r*_OH_ ∈ [0.8–1.5] Å, *r*_HH_ ∈ [1.0–2.5] Å). Other intramolecular GAPs employed SOAP descriptors with cut-offs shown outlined in the figure captions. Intermolecular GAPs were trained by subtracting intramolecular energies and forces of all the defined components from the reference total energy. Potentials for all pure condensed phase systems were trained and applied at or close to their experimental liquid densities using 10 solvent molecules (*e.g.* 10 H_2_O molecules in a 343 Å^3^ cubic box). Aqueous Zn and explicitly solvated S_N_2 reactions used 20 water molecules in a cubic box with side length of 10 Å. All files required to reproduce the examples shown here can be found in https://github.com/duartegroup/gap-train/tree/master/examples/paper_examples.

GAP-MD simulations were performed with ASE^78^ interfaced to QUIP with the quippy wrapper using the Langevin integrator with 0.5 fs timesteps at 300 K unless otherwise specified. Condensed phase MD simulations were performed with three-dimensional periodic boundary conditions following minimisation and equilibration for at least 20 ps. Initial configurations, CUR^61^ selection and all learning curves were generated with the *gap-train* module, which was used to run the automated fitting.^79^ All active learning was performed using a ‘diff’ strategy, where a configuration is added to the training set if |*E*_0_ − *E*_GAP_| is larger than a threshold. With system-dependent hyperparameter optimisation, using a threshold on the maximum atomic variance predicted by the Gaussian process (‘gp_var’) can result in accelerated learning (Fig. S25†). CUR selection used SOAPs averaged over atoms in a configuration using the Dscribe^80^ package (‘inner’ averaging, over entries of the expansion coefficient vector). Intra + inter (I + I) energy and force evaluations used an expansion factor of 10 to ensure no intermolecular atoms were within the intra GAP cut-off. The NumPy^81^ based implementation introduces a negligible computational overhead for expanding the box (∼0.1 ms per step real time) but requires two GAP calculations on the inter and intra components, currently carried out in serial. All generated potentials, with the exception of the revPBE0-D3 water potential and metallocage, were trained in less than a day on 10 CPU cores. The revPBE0-D3 water potential was constructed without any prior data in 5 days (1 intra + 4 inter) and used 20 CPU cores, while the metallocage fragment was trained for 3 days also on 20 CPU cores. Explicit S_N_2 reaction dynamics simulations were performed using intra components for H_2_O and [Cl⋯CH_3_Cl]^−^, where the latter, due to the finite atomic cut-off employed, has the correct dissociation behaviour when Cl^−^ and CH_3_Cl are distant.

Periodic DFTB calculations performed with DFTB+^82^ using 3ob^83^ parameters, and molecular equivalents using GFN2-XTB^84^ in XTB v. 6.2.3. Periodic pure DFT calculations were performed with GPAW^85,86^ v. 19.8.1 with the PBE^87^ functional and a 400 eV plane-wave cut-off from a *dzp* LCAO initial guess at the gamma point. Hybrid periodic DFT calculations with the revPBE0^88,89^ functional combined with the D3^90^ dispersion correction were performed with CP2K.^91^

Molecular DFT, MP2 and coupled cluster [DLPNO-CCSD(T)] calculations used for training were performed with ORCA^92,93^ v. 4.2.1 wrapped with autodE^94^ using PBE^87^ and PBE0^89^ functionals, (ma)-def2-SVP, def2-TZVP and ma-def2-TZVPP basis sets.^95^ AIMD calculations at the DFTB level were performed with DFTB+ with 3ob parameters^83^ and MM simulations were carried out with GROMACS^96,97^ 2019.2 with TIP3P parameters.^98^

To evaluate *τ*_acc_ on the fully reactive water NN of Cheng *et al.*,^36^ the NN was retrained on 7258 configurations from ref. 99, which were re-evaluated at DFTB(3ob) and trained using n2p2^100^ using the same parameters and symmetry functions.

## Data availability

All files required to use this method and reproduce the examples presented in this manuscript can be found at https://github.com/duartegroup/gap-train/.

## Author contributions

TAY developed and implemented the strategy, and carried out the calculations. TJW contributed to development and implementation of the strategy, and to MM-MD calculations for training. All authors participated in data analyses. TAY and FD conceptualised the study. FD and VLD supervised the study and wrote the manuscript with TAY.

## Conflicts of interest

There are no conflicts to declare.

## Supplementary Material

SC-012-D1SC01825F-s001
