# Supplementary material for: A transferable active-learning strategy for reactive molecular force fields
Source: Chem Sci. 2021 Jul 5;12(32):10944–55. doi: 10.1039/d1sc01825f (PMC8372546; doi:10.1039/d1sc01825f)
Supplement: SC-012-D1SC01825F-s001 [file SC-012-D1SC01825F-s001.pdf]

Electronic Supplementary Material (ESI) for Chemical Science.
This journal is © The Royal Society of Chemistry 2021

Supporting information:

A transferable active-learning strategy for reactive molecular force fields

Tom A. Young,[a] Tristan Johnston-Wood,[a] Volker L. Deringer,[b] Fernanda Duarte[a]

[a] Chemistry Research Laboratory, University of Oxford, Mansfield Road, Oxford OX1 3TA, United Kingdom

[b] Department of Chemistry, Inorganic Chemistry Laboratory, University of Oxford, Oxford OX1 3QR, United Kingdom

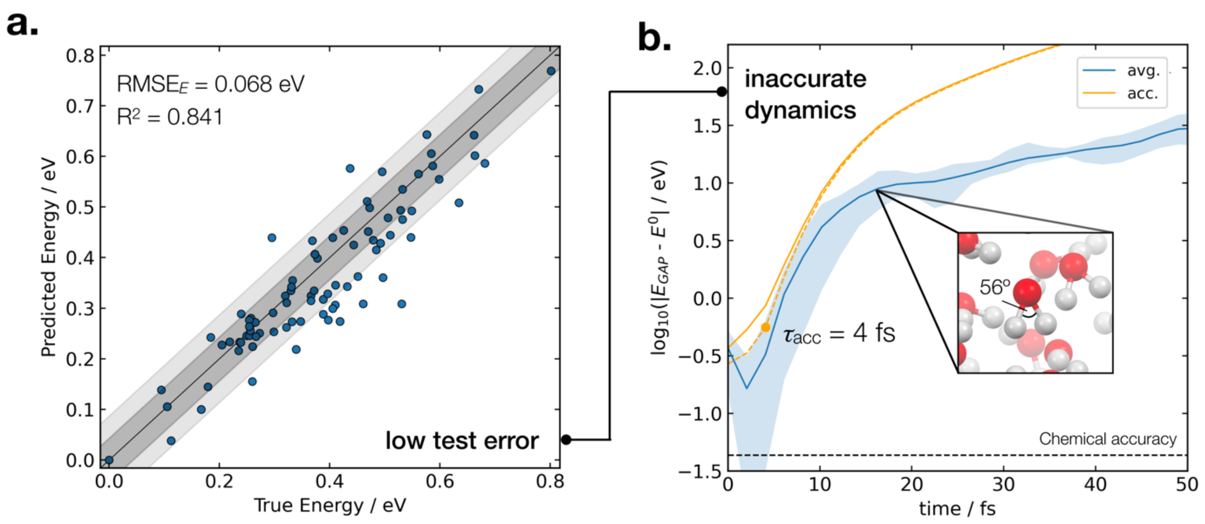

Figure S1. (a) A GAP model for molecular water (fitted using the P1 parameter set, **Table S1**) as characterized by its accuracy on external validation data not encountered in training; dark and light shaded area bound the ‘chemically accurate’ ±1 kcal mol^{-1} and ±2 kcal mol^{-1} regions, respectively. (b) The resulting difference between predicted and true single point energies on MD frames at 300 K ($\delta t = 0.5$ fs). Dynamics with 30 water molecules in a cubic box ($l = 10$ Å, $\rho \sim 1$ g cm^{-3}). Error range (min–max, shaded) and average of five simulations using the same trained GAP from different initial randomly placed then minimized points. DFTB(3ob) ground truth. Orange lines depict the total cumulative error (solid) and cumulative error above 0.1 eV (dashed).

Table S1. Parameter sets for GAPs, SOAPs and 2/3b descriptors.

Set	Type	Parameter	Value
P1	GAP	σ_E	0.316 meV
		σ_F	0.1 eV Å^{-1}
		ζ	4
	2b descriptors: O–H, H–H, O–O	r_c	5.5 Å
		n_{sparse}	30
		δ^{2b}	1.0 eV
		θ	1.0 Å
		sparse method	Uniform
	SOAP descriptor: O	r_c	3.0 Å
		n_{sparse}	100
		δ^{SOAP}	0.1 eV
		σ_{at}^{SOAP}	0.5 Å
		n_{max}, l_{max}	6
		sparse method	CUR points
P2	GAP	σ_E	0.316 meV
		σ_F	0.1 eV Å^{-1}
		ζ	4
	SOAP descriptors	r_c	DFTB: 3.0 Å<br>PBE: 3.5 Å<br>rPBE0: 4.0Å
		n_{sparse}	500
		σ_{at}^{SOAP}	0.5 Å
		n_{max}, l_{max}	6
		sparse method	CUR points

Table S2. Outline of training strategies used to train a GAP for bulk water (**Figure 1**). N total ground truth evaluations used to train the final potential. Errors are quoted as standard errors in the mean from 5 independent samples where appropriate to one significant figure. All training used 10 water molecules in a cubic box with side length 7 Å.

	**Strategy**	**Notes**	τ_{acc} / ps	N
1	MM-MD	Classical molecular mechanics MD simulations were performed at 100, 300, 500 and 1000 K using GROMACS v. 2019.2 with a timestep of 1 fs and TIP3P water. Following a 1 ns NVT equilibration of a random configuration of water 10 ns of NVT dynamics were performed taking 1000 evenly spaced frames from the simulation.	0	1000
2	rand.	Configurations were generated by adding GFN2–XTB optimised water molecules into the box in a random position and orientation, ensuring that there are no intermolecular distances < 1.5 Å. Random displacements were added to each Cartesian coordinate sampled from a random normal distribution with σ=0.05 Å, which samples over intramolecular bond stretches and bends.	0	1000
3	rand. min.	As *rand.* where each random configuration is minimized to $\|\boldsymbol{F}_i\| < 1$ eV $Å^{-1}$, where $\boldsymbol{F}_i$ is the force on atom i. Subsequently, random normal displacements were added to each atom to ensure some sampling of the intramolecular modes.	0.0008 ± 0.0007	7490 ± 20
4	AIMD	*Ab-initio* MD simulations were performed at the ground truth level (DFTB, 3ob parameters) for 1 ps at 300 K with a 0.5 fs timestep. Frames were randomly selected from the trajectory with at least a 2 fs interval.	0.011 ± 0.003	2570 ± 10
5	AL	Active learning is initiated from a GAP trained on 10 random configurations with a 1.5 Å minimum distance between water molecules. MD simulations at 300 K was then propagated using this potential for $n^3 + 2$ fs, where n is the number of iterations of the MD trajectory. The error between the ground truth (E_0) and predicted energy (E_{GAP}) is evaluated for the final frame ($\|E_0 - E_{GAP}\|$) and, if above 0.1 eV the configuration is added to the training data. If above 10× the threshold then the error is backtracked in intervals of 2 fs until a suitable configuration is obtained, as to not add any very high energy configurations. If the error is 100× the threshold, then the first frame of the trajectory is returned, as the backtracking is likely to be too slow. If the error is below the threshold, then a further MD trajectory is propagated, and n incremented by one. If $n > 10$ then no configuration is added from this set of trajectories.	0.07 ± 0.02	3200 ± 200
6	AL-I+I	Intra- and intermolecular interactions were trained independently, and separate GAPs trained for each term. The intramolecular GAP is trained on a cubic grid of configurations $r_{O\text{-}Ha}$, $r_{O\text{-}Hb} \in [0.8, 1.5]$ Å, $r_{Ha\text{-}Hb} \in [1.0, 2.5]$ Å with 8 points in each dimension with 2- and 3-body descriptors with 3 Å cut-offs and 30 sparse points. The intermolecular GAP is trained using the active learning method outlined above. Energies and forces were calculated as a sum of terms and the intramolecular GAP prediction evaluated in a box expanded by a factor of 10 while maintaining the fractional coordinate of the centre of mass of each water molecule fixed, as to ensure no intermolecular hydrogens are in the radius of the intramolecular descriptors.	31 ± 7	1160 ± 90

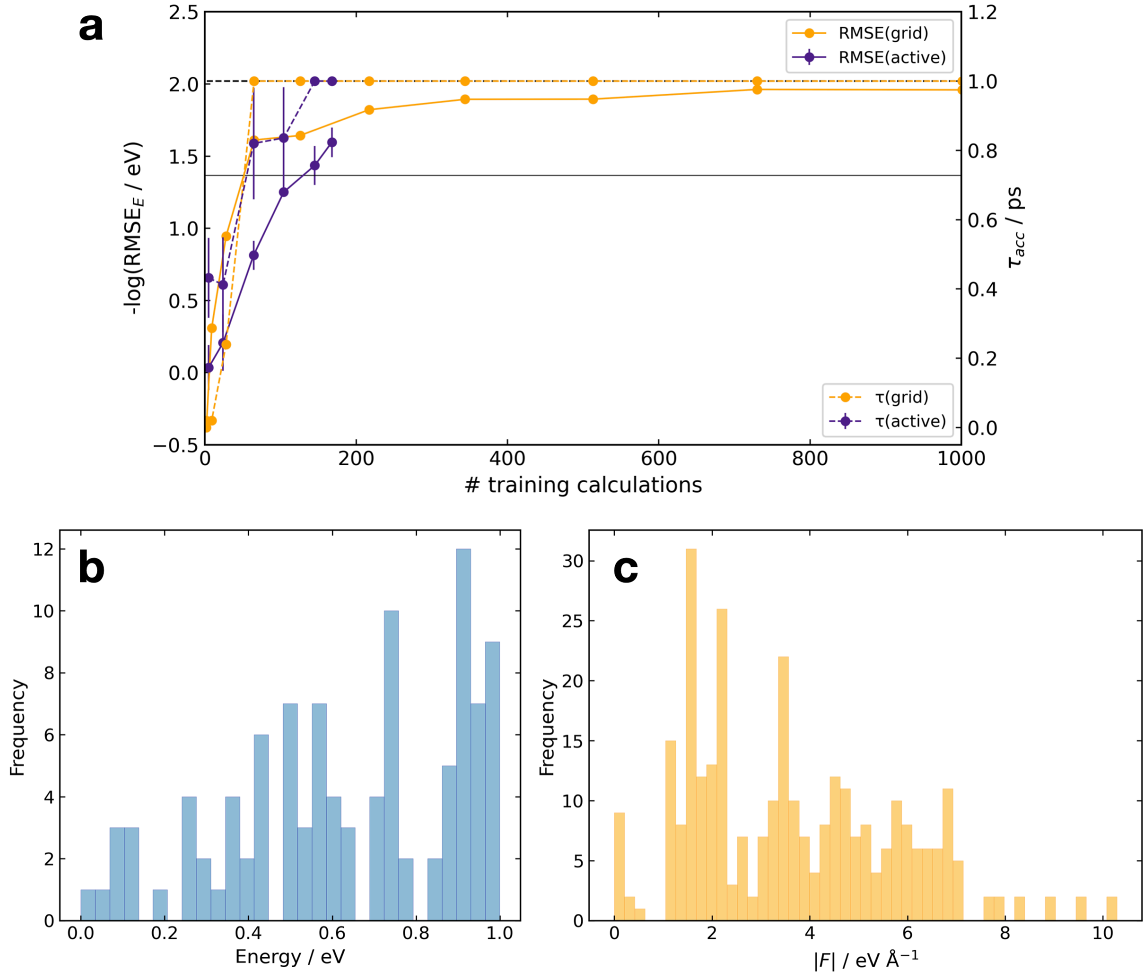

Figure S2. (a) Comparison of active learning and a grid-based approach for training a water monomer. 2b+3b GAP with r_c = 3.0 Å without a SOAP all other parameters as **P2** (**Table S2**). max(τ_{acc}) = 1 ps calculated in a 2500 K simulation for a ~1% probability of accessing a configuration 1 eV above the minimum, E_l = 0.043 eV, E_t = 0.43 eV. (b, c) Energy and force distribution of the test data used to calculate a root mean squared error (RMSE) generated on a grid over r_{OHa}, r_{OHb} ∈ [1.0, 1.3] Å and r_{HaHb} ∈ [1.0, 2.5] Å and truncated above 1 eV of the minimum to 103 datapoints that are not coincident with any training data.

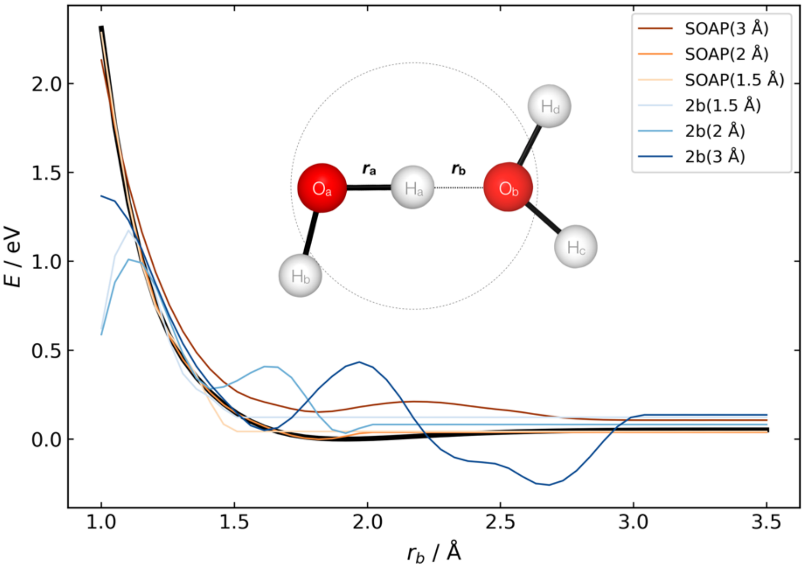

Figure S3. Water dimer PES predicted using SOAP and 2b GAPs with the ground truth (DFTB(3ob)) in black. Trained on then evaluated on the PES points. Intramolecular component subtracted using the same intra-GAP as **Figure 1** (2b+3b, r_c=3.0 Å) evaluated in separate boxes.

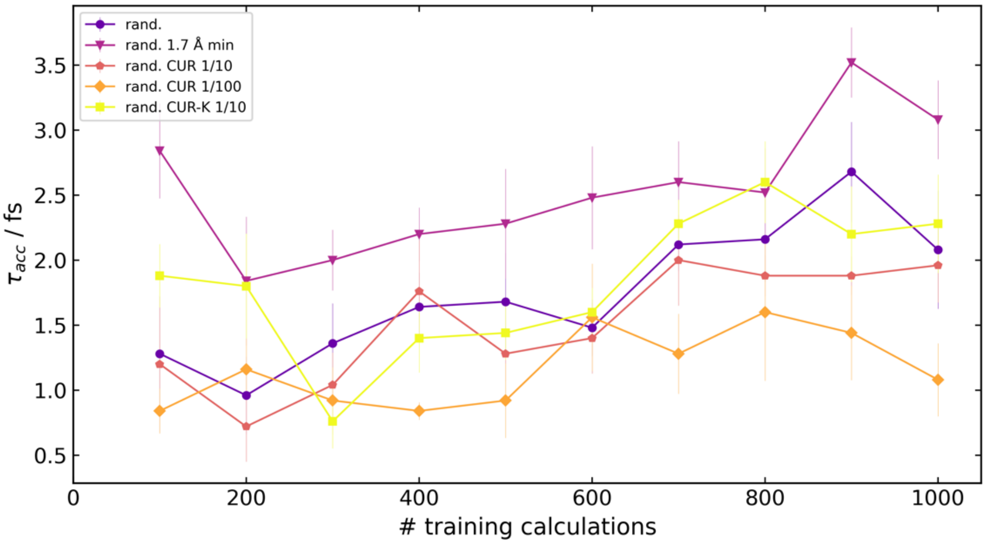

Figure S4. Learning curves for a bulk water GAP trained on random configurations, with or without selection strategies. Water molecules randomized in the box by applying a random rotation and translation to each water molecule ensuring no intermolecular distance < 1.5 Å, apart from *rand. 1.7 Å min* (purple triangles) where the minimum distance is 1.7 Å. τ_{acc} calculated with a 1 fs interval, E_l = 0.1 eV, E_t = 1 eV averaged over 5 initial random configurations. Error bars are standard error in the mean over 5 independent iterations. *CUR-K 1/10* is a CUR selection of the square kernel matrix between SOAP descriptors calculated using Dscribe,[1] keeping 1 in 10 rows, *CUR* is selection on the SOAP matrix averaged over atoms in the box.

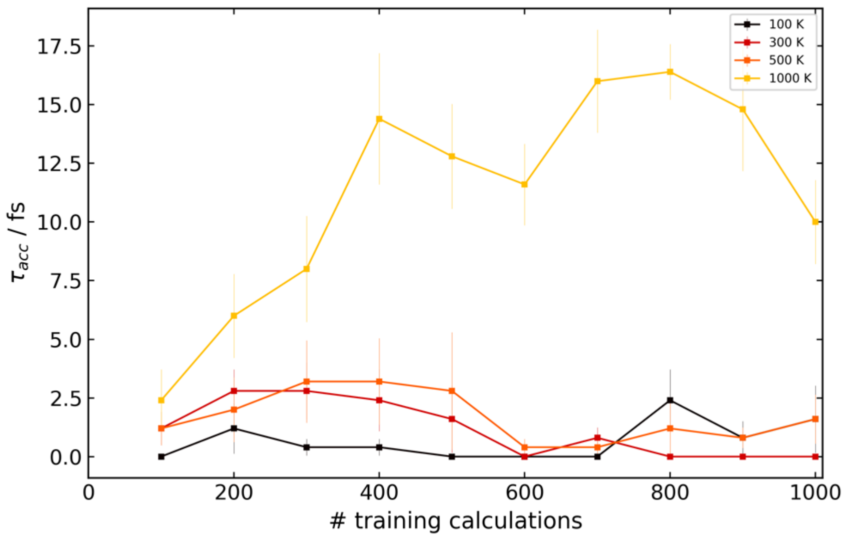

Figure S5. Learning curves for a bulk water GAP trained on classical molecular mechanics configurations at different temperatures. Initial random configuration minimized then equilibrated for 1 ns, TIP3P parameters, flexible water. Configurations taken evenly spaced from a total of 10 ns of simulation time. τ_{acc} calculated with a 10 fs interval, $E_l = 0.1$ eV, $E_t = 1$ eV averaged over 5 initial random configurations. Error bars are standard error in the mean over 5 independent iterations.

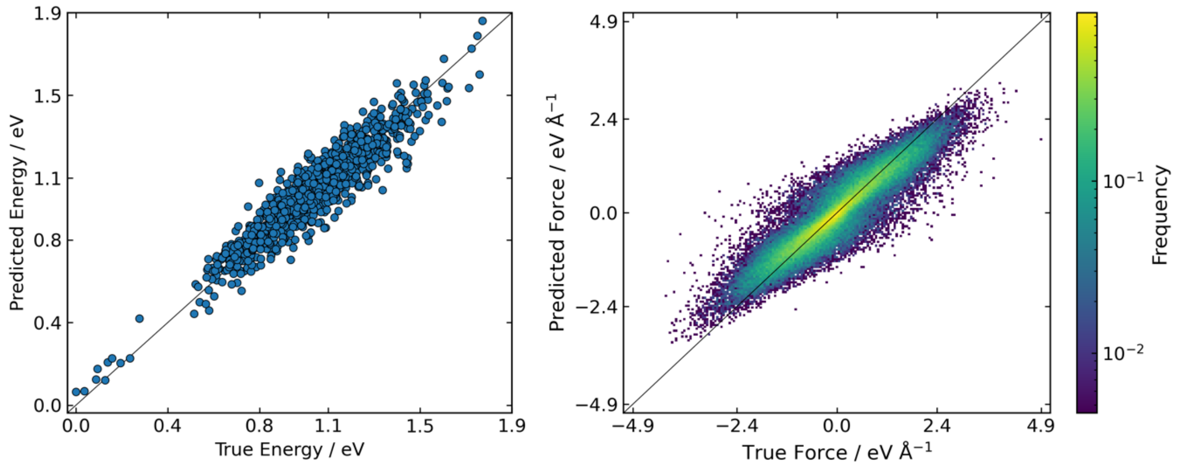

Figure S6. Correlation plot between predicted and 'true' (DFTB) energies and forces on MM training data (300 K) for bulk water (TIP3P parameters).

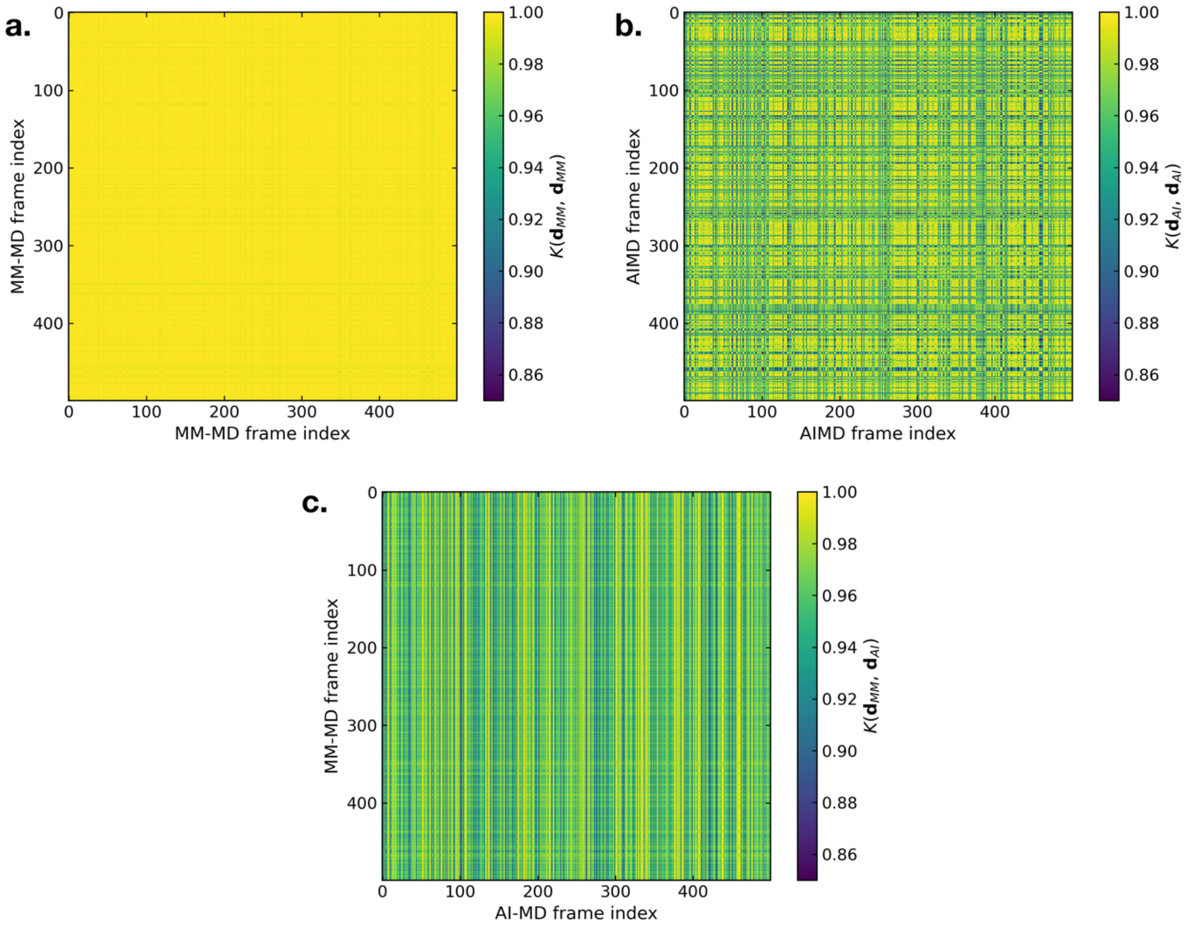

Figure S7. Kernel matrices on normalised SOAP vectors between MM-MD and AIMD frames simulated at 300 K calculated using Dscribe,[1] with 'inner' averaging (average coefficients over sites before summation over angular projection, m) over unique elements (H, O), r_c =5 Å, n_{max}=l_{max}=6, raised to the 4th power (i.e. ζ=4). Values 0–1 correspond to minimal–maximal similarity in configurations.

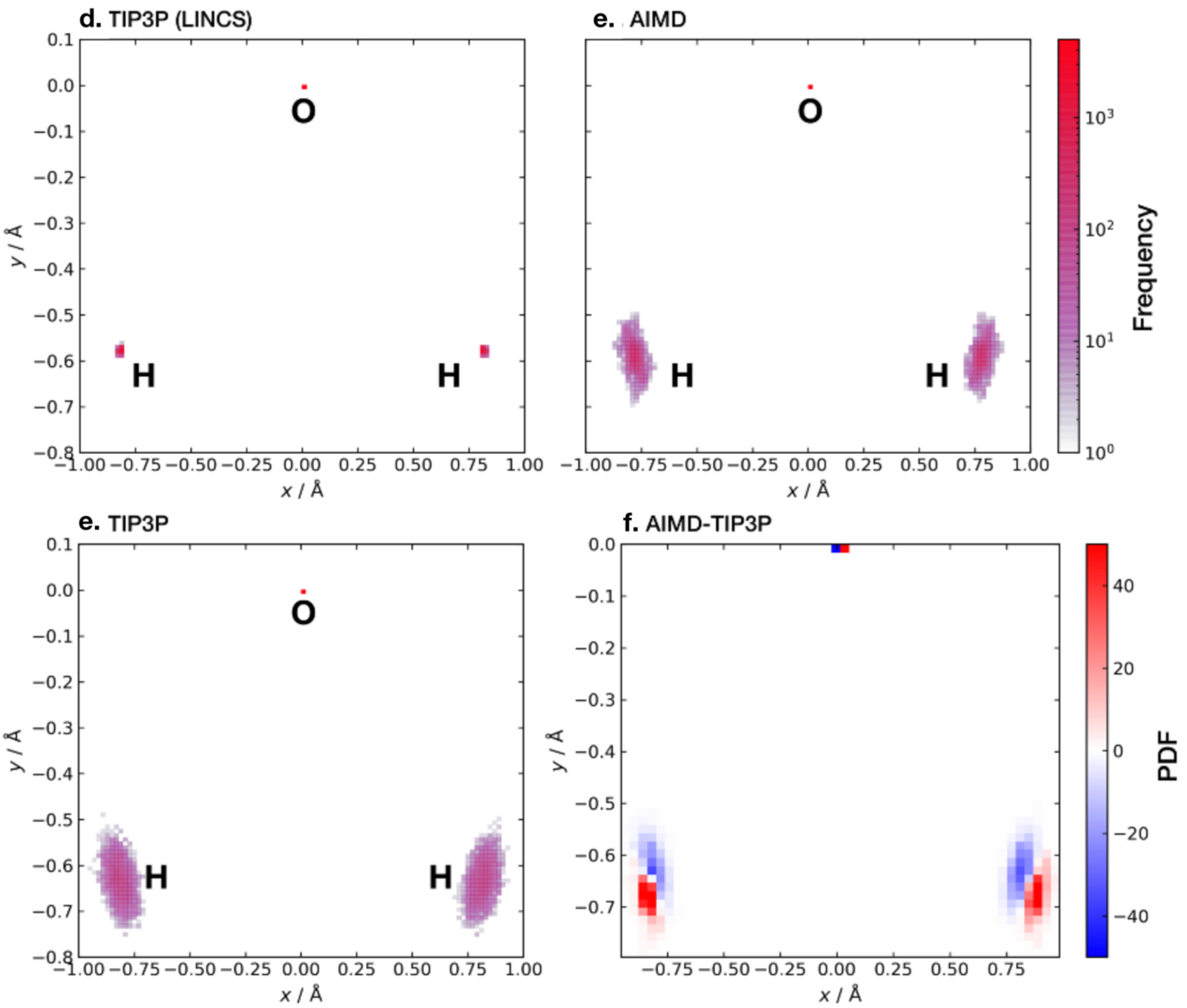

Figure S7 cont. Histogram of atomic positions in (a) MM(TIP3P) and (b) AIMD(DFTB) configurations generated over a 300 K trajectory. All water molecules fitted to an initial reference in the *xy* plane with the Kabash algorithm, oxygen centred at (0, 0). Only molecules wholly within the box included.

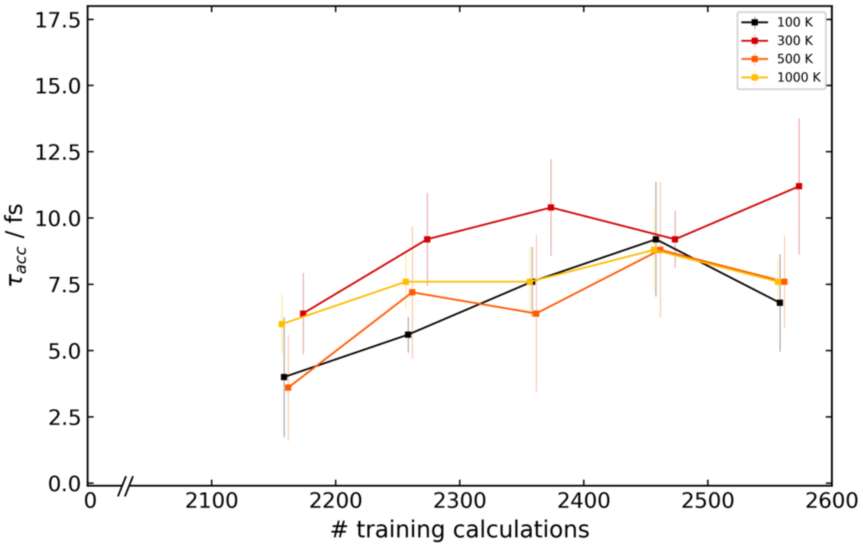

Figure S8. Learning curves for a bulk water GAP trained on classical 'ab-initio' [AIMD, DFTB(3ob)] configurations at different temperatures. Initial random configuration minimized at DFTB. Configurations taken evenly spaced from a total of 1 ps of simulation time. τ_{acc} calculated with a 10 fs interval, $E_l = 0.1$ eV, $E_t = 1$ eV averaged over 5 initial random configurations. Error bars are standard error in the mean over 5 independent iterations.

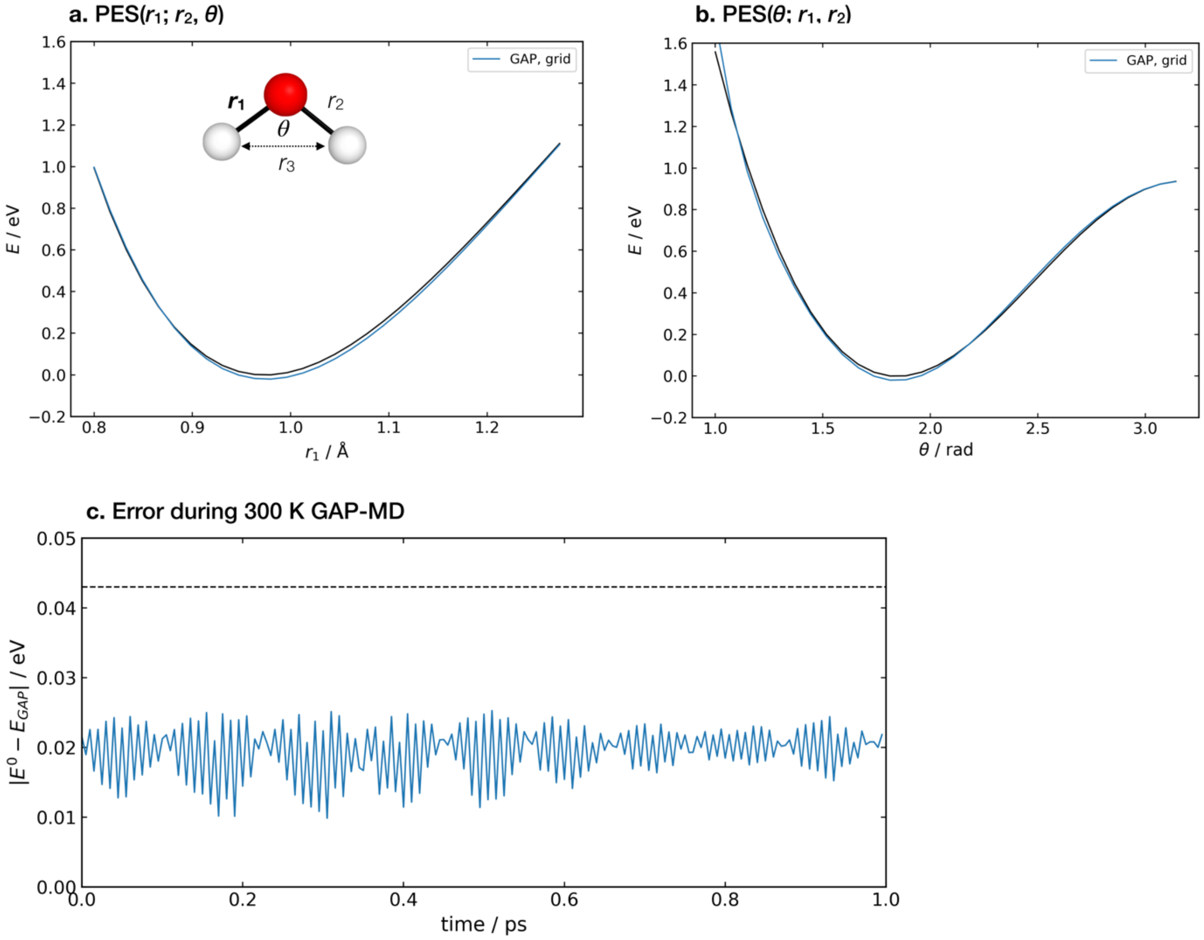

Figure S9. (a, b) Potential energy surfaces and (c) error in gas phase monomer dynamics generated by GAPs trained on 65 data points in a grid over r_1, $r_2 \in [0.8, 1.5]$ Å, $r_3 \in [1.0, 2.5]$ Å including the minimum at the DFTB(3ob parameters) level of theory. Two-body + three body GAP trained with 3.0 Å cut-offs.

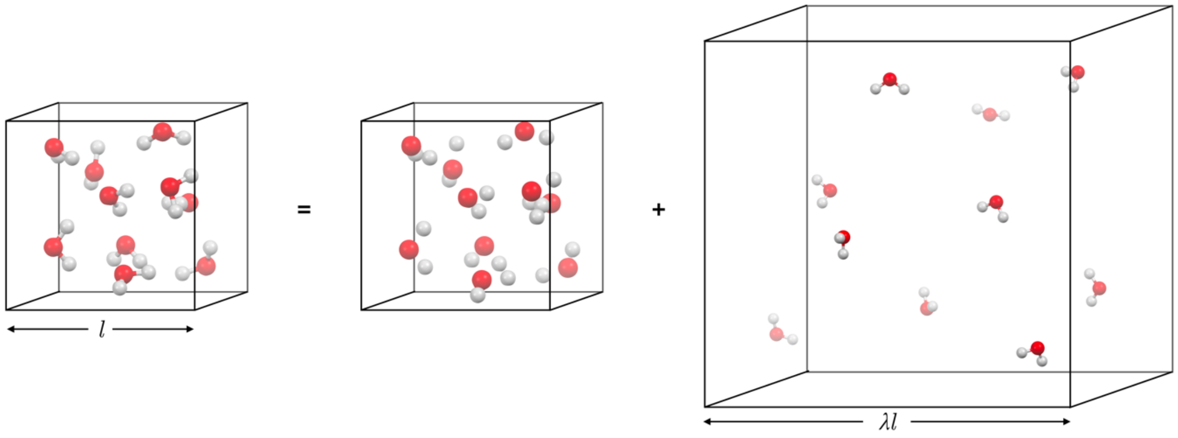

Figure S10. Schematic representation of an intra+inter (II) energy evaluation using two GAPs. Total energy is a sum of the intermolecular interactions in a box, plus the intramolecular energy calculated by expanding the box by a factor λ, keeping the fractional centre of masses of each molecule fixed.

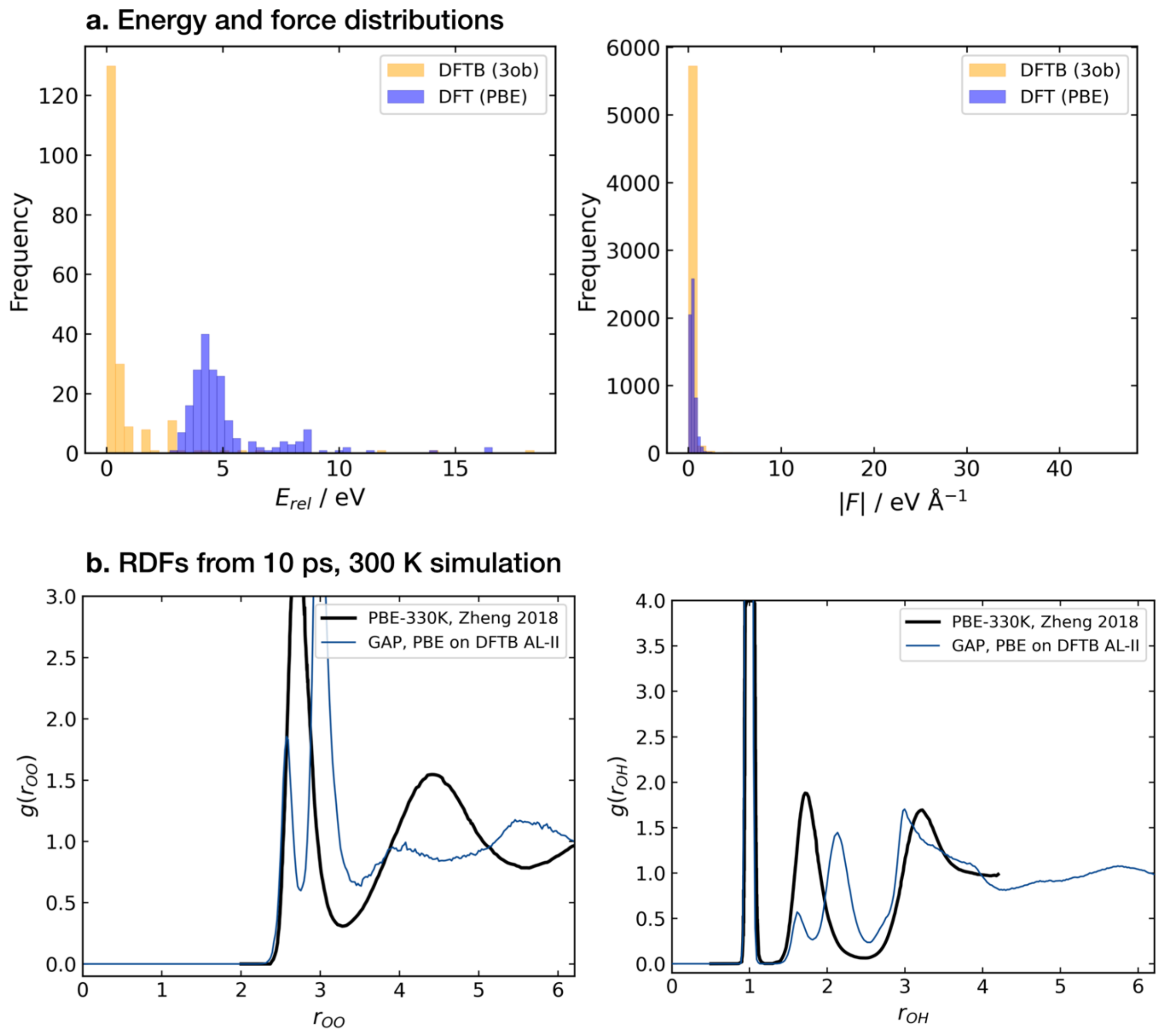

Figure S11. Attempted DFT (PBE/400 eV) uplift of DFTB GAP with single point energy and force evaluations on a set of configurations used to train a stable inter GAP (AL-II, **Figure 1**). Intramolecular energy evaluated using a DFT GAP trained using active learning at 1600 K with a 2 eV maximum energy threshold. (a) Energy and force histograms, where the DFT configurations are referenced to the lowest energy located in an active learning cycle at the DFT level. (b) Pair radial distribution functions calculated from a 10 ps, 300 K MD simulations with the inter (+intra) GAP trained on the DFTB configurations, compared to a PBE reference from ref. 2.

S1. Other solvents

To demonstrate the transferability of the intra+inter active learning training method to other solvents presented here are learning curves in τ_{acc} for a selection of small, commonly used, organic solvents. The accuracy of the potential is quantified by the final error metric following the full active learning, and as a quick validation the radial distribution functions are shown for all pairs in each system. Given the much higher dimensionality of the intramolecular PES in all of the solvents a dense grid over all coordinates is not possible (e.g. $N_{atoms} = 6$ in MeCN $\Rightarrow 8^{(3\times6)-6} \sim 10^{11}$ points) and active learning needs to be employed for the intra surface. Employing active learning on the intramolecular modes of water requires both a high temperature, as to sample the curvature of the PES at regions often sampled at 300 K, and an energy threshold to prevent high energy configurations entering the fit (**Figure S13**).

Active learning at 1600 K then readily dissociates Cl• with a DFTB ground truth.

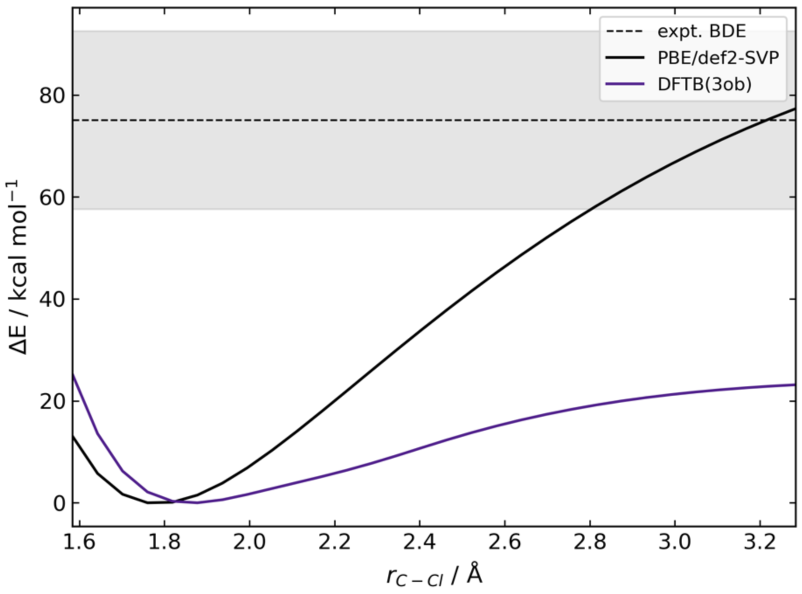

Figure S12. Comparison between DFT (PBE/def2-SVP) and DFTB energies for the C–Cl dissociation curve in CH_2Cl_2. Geometries from unrelaxed PES scan from the DFT geometry. Experimental data indicated with dash grey line from ref. [3].

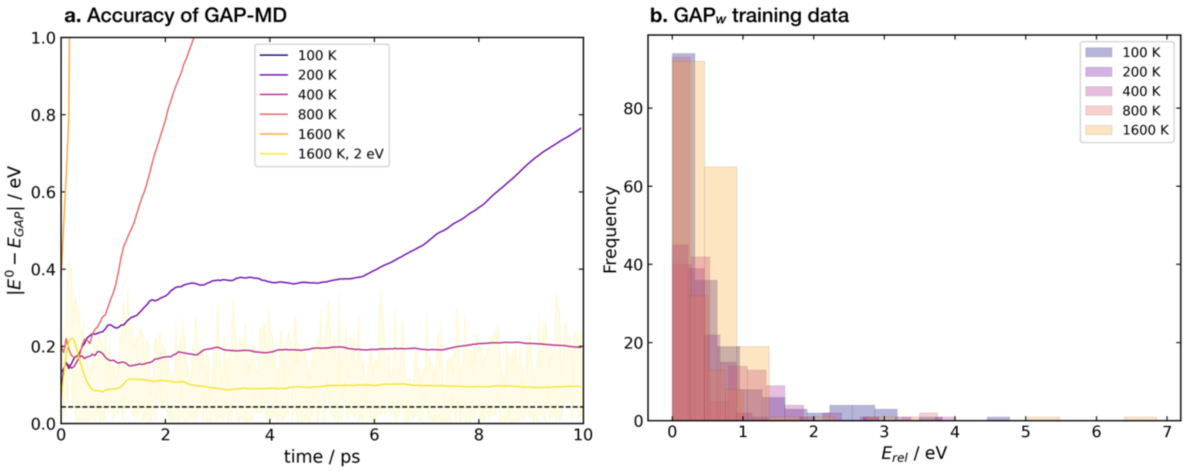

Figure S13. Absolute errors between GAP and the ground truth DFTB on GAP-MD trajectories propagated at 300 K of a water box. Intramolecular GAP(2b+3b) trained using active learning using intermediate GAP-MD at the quoted temperature. *1600 K, 2 eV* also specifies an energy cut-off on GAP training data. Intermolecular GAP trained as **Figure 1** (AL-I+I). Error ranges are generally not shown for clarity. Initial random configurations to start the active training loop are identical for each training run.

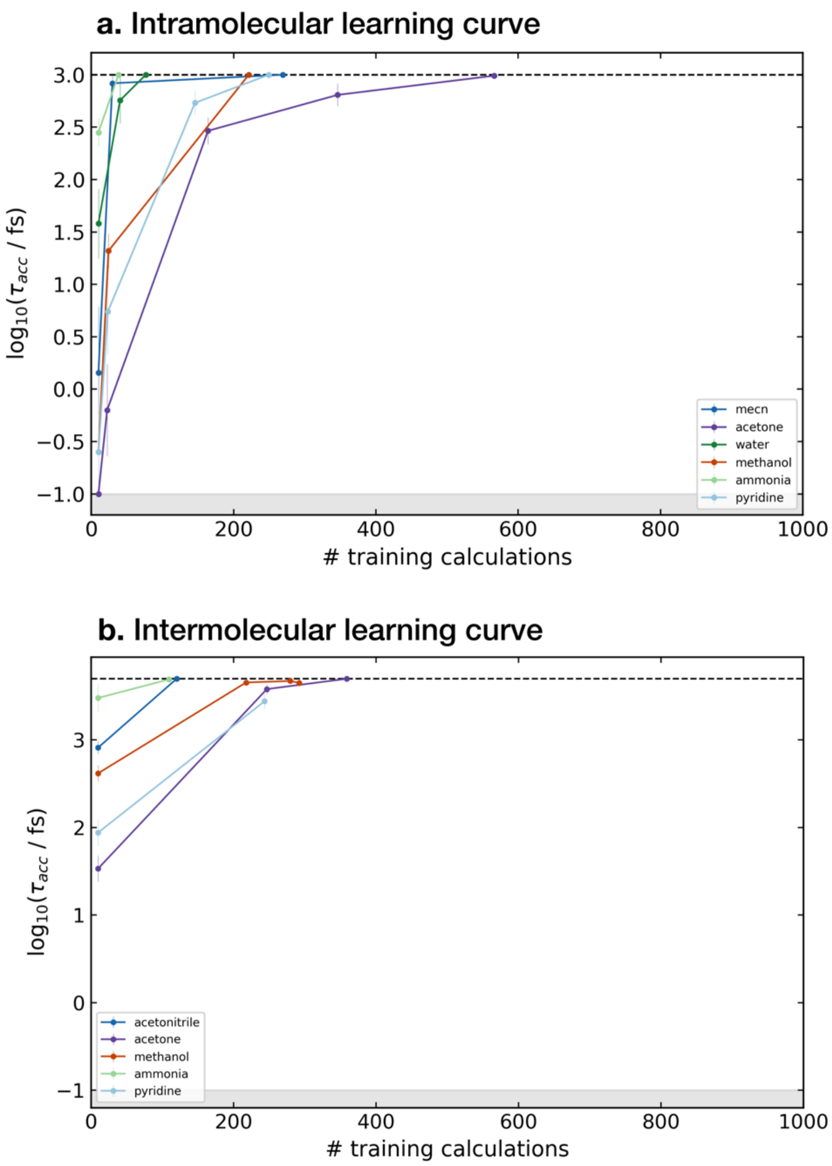

Figure S14. Learning curves (a) intra- and (b)intermolecular components in different molecular solvents. Parameters for each system shown in **Table S3**. 1600 K for all intramolecular active learning and 300 K for the intermolecular equivalent from 10 initial random normal displacements of all atoms (σ = 0.05 Å, μ = 0 Å). Maximum τ_{acc} shown as dashed lines and min(τ_{acc}) = 0.1 fs for plotting. Error bars plotted as the standard error in the mean from 5 independent repeats. Active learning halted if no configurations have error above the threshold.

Table S3. Box sizes for intermolecular training and SOAP descriptors used for GAPs shown in **Figure S14**. All intermolecular training used 10 molecules initially optimised at the GFN2-XTB level of theory. Box size chosen to ensure *P*(generated) ⪆ 0.1 when molecules are added to the box ensuring a minimum distance of >2 $\times X_{\text{VdW}}$ – 0.5 Å where X_{VdW} is the van der Waals radius of the largest atom (X) in the system. All descriptors used r_c^{SOAP} = 3 Å and other parameters as **Table S1**. SOAP descriptors shown as e.g. X: Y, Z, as a SOAP on atom type X which includes other types Y and Z. 0.04 eV ~ 1 kcal mol^{-1}.

Molecule	Inter/intra	Box length / Å	Descriptors	E_l / eV	E_t / eV	max(τ_{acc}) / ps
Acetonitrile	intra	10	C: H, C, N	0.043	0.43	1
	inter	13	C: H, C, N<br>N: H, C, N	0.1	1.0	5
Acetone	intra	10	C: H, C, O	0.043	0.43	1
	inter	15	C: H, C, O<br>O: H, C, O	0.1	1.0	5
Water	intra	10	O: H	0.043	0.43	1
Methanol	intra	10	C: H, C, O<br>O: H, C, O	0.043	0.43	1
	inter	12	C: H, C, O<br>O: H, C, O	0.1	1.0	5
Ammonia	intra	10	N: H	0.043	0.43	1
	inter	11	N: H, N	0.1	1.0	5
Pyridine	intra	10	C: H, C, N	0.043	0.43	1
	inter	15	C: H, C, N<br>N: H, C, N	0.1	1.0	5

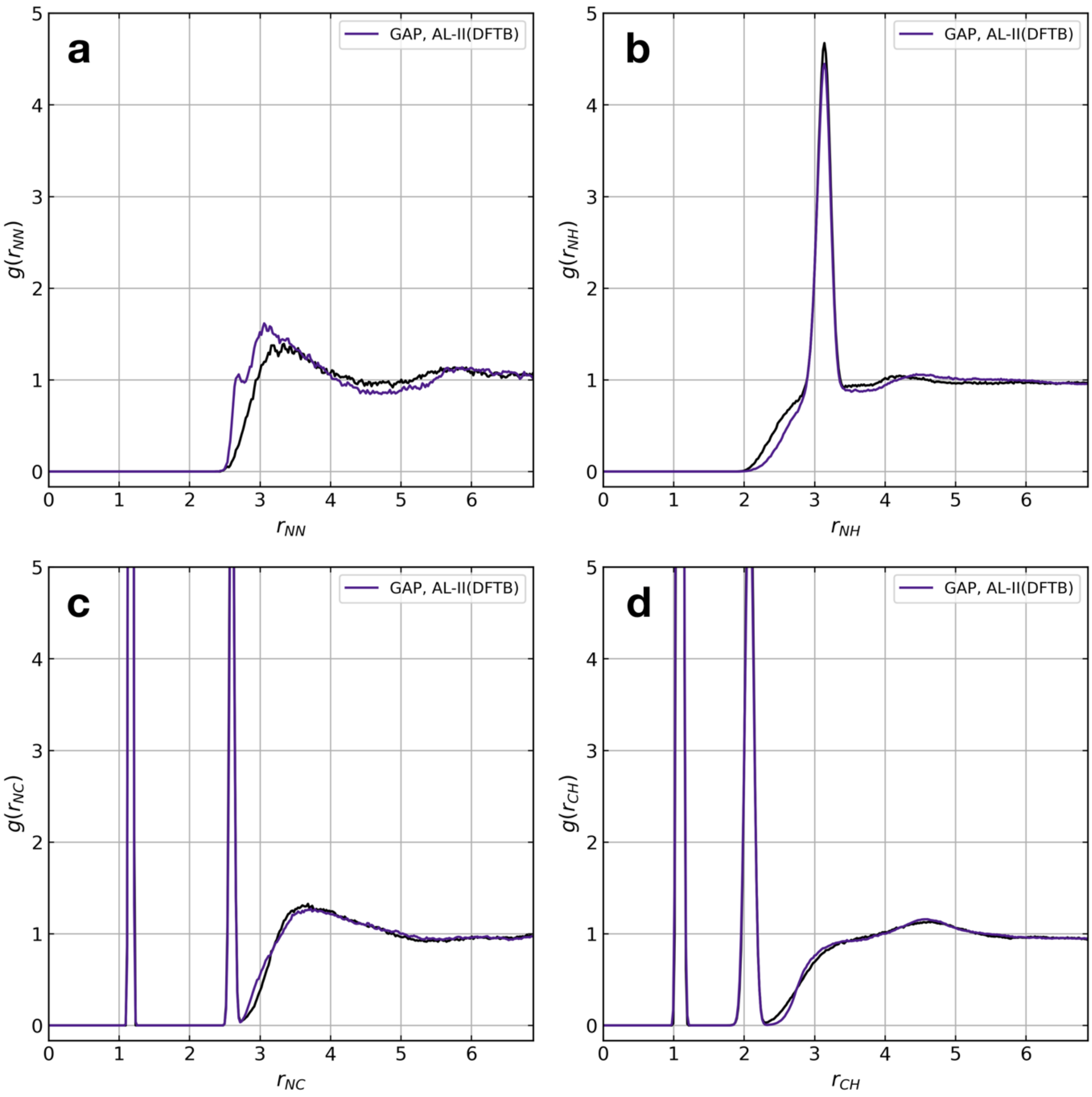

Figure S15. Radial distributions functions for acetonitrile generated using GAPs (purple) trained with active learning on inter and intramolecular degrees of freedom (as **Figure S14**) ground truth DFTB(3ob) level (black). Dynamics run for 30 ps at 300 K in a 13.8 Å length box with a time-step of 0.5 fs.

S2. Reactions

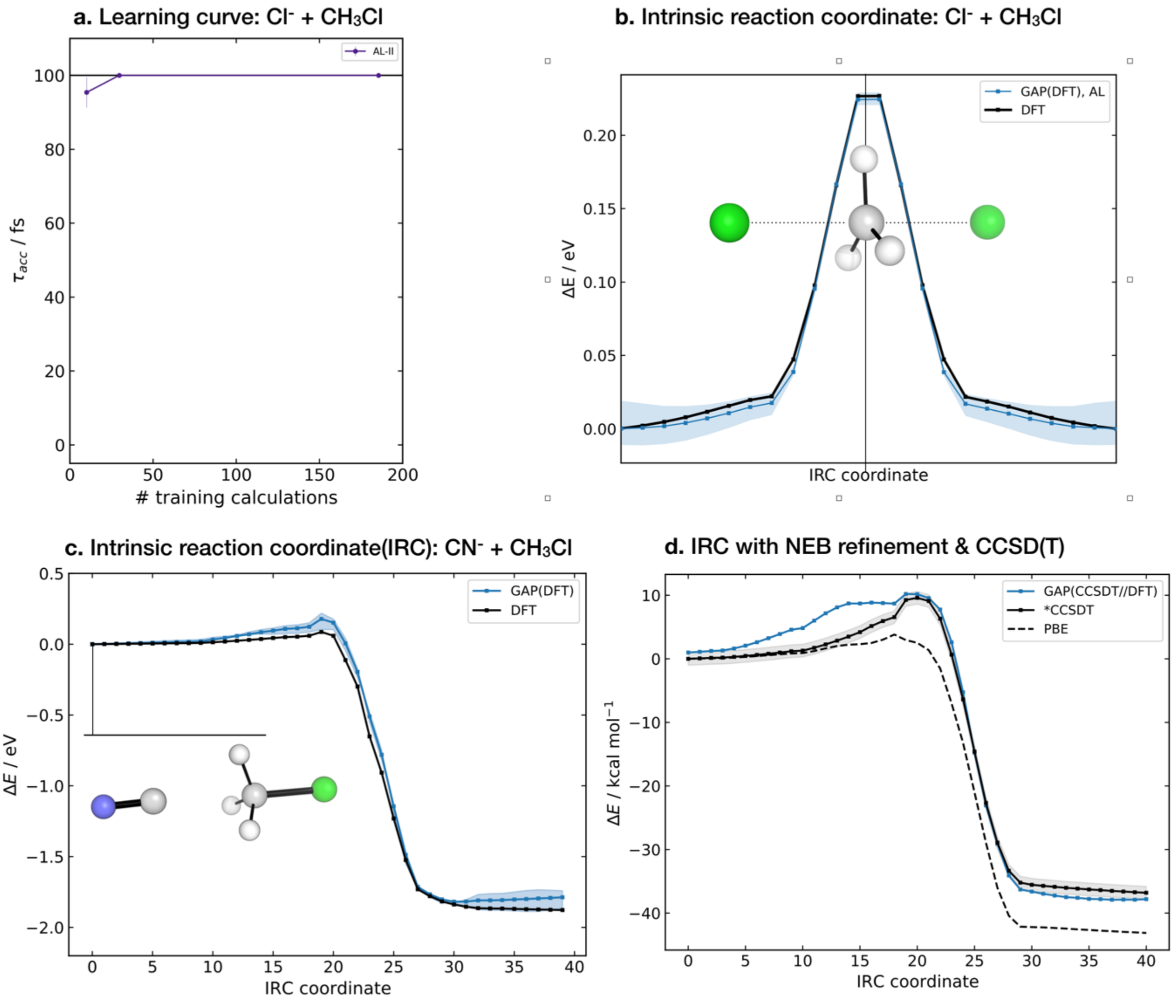

Figure S16. (a) Learning curve for TS originated dynamics in the gas phase for $Cl^- + CH_3Cl \rightarrow Cl^- + CH_3Cl$. Number of ground truth evaluations does not include those used to find the initial transition state. SOAP descriptors with $r_c = 3.5$ Å on C and Cl. TS optimisation and energy/force evaluations performed with ORCA at the PBE/ma-def2-SVP level of theory. τ_{acc} calculated using a 2 fs time interval, 1 kcal mol^{-1} error threshold, 10 kcal mol^{-1} maximum total error to a maximum of τ_{acc} = 100 fs, as only short time dynamics are required from the TS. (b) Intrinsic reaction coordinate (IRC) for $Cl^- + CH_3Cl \rightarrow Cl^- + CH_3Cl$ calculated in ORCA at the ground truth (PBE/ma-def2-SVP) and predicted with 5 trained GAPs. Average shown as the blue line and the range of predictions in blue. IRC configurations were not present in the training data. (c) As (b) for $CN^- + CH_3Cl \rightarrow Cl^- + CH_3CN$ but trained using uphill active learning i.e. without knowledge of the TS. 0.5 eV of energy was added to the breaking C–Cl bond and dynamics propagated for up to 500 fs at a temperature of 200 K. (d) Predicted IRC using the active-learnt GAP with configurations added from close to the minimum energy pathway with NEB relaxation (final geometry selected manually) using the GAP, energies and forces calculated, then re-predicted. Coupled cluster single point energies and numerical frequencies were then calculated on the 200 configurations and the IRC compared. *CCSD(T) ≡ DLPNO-CCSD(T)/ma-def2-TZVPP) energy values on the MP2/ma-def2-TZVPP.

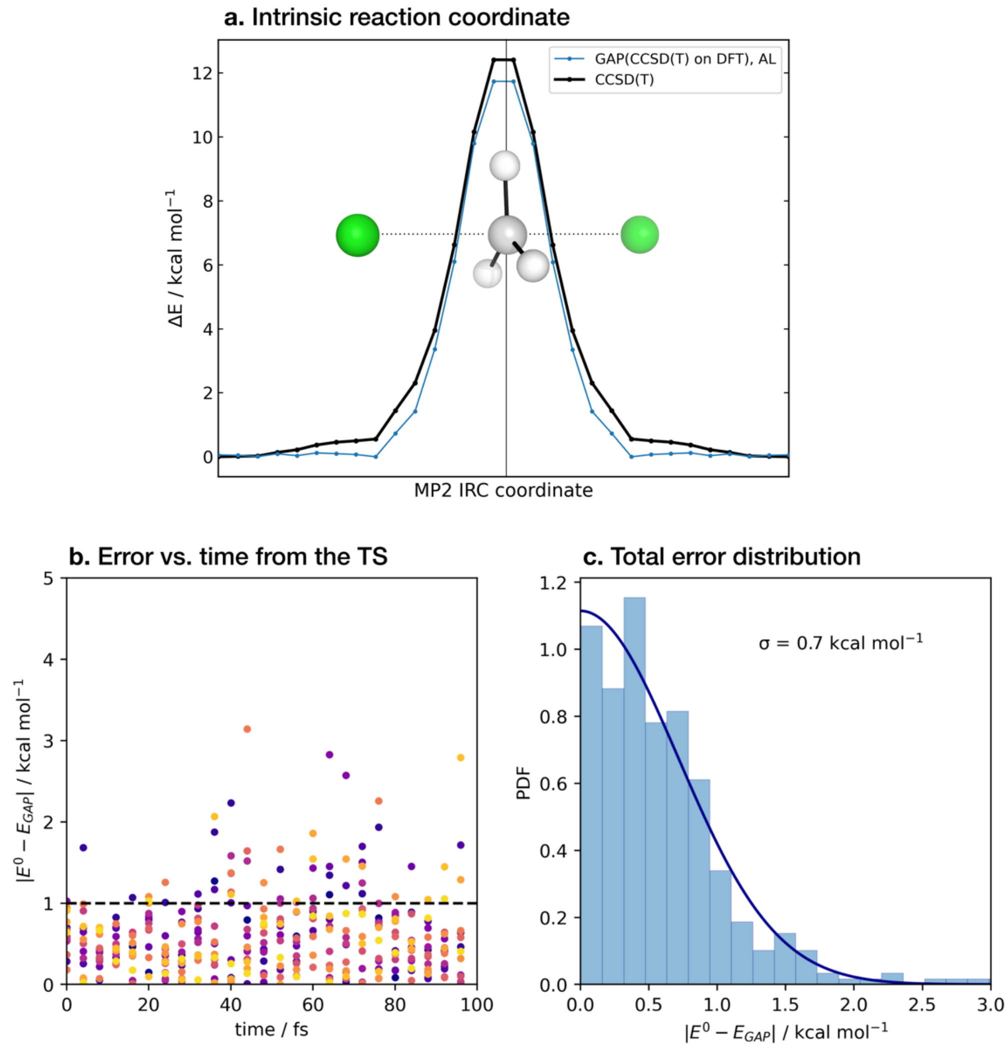

Figure S17. (a) Accuracy of a GAP trained on configurations generated from DFT (PBE/ma-def2-SVP) active learning (see **Figure S16)** for $Cl^- + CH_3Cl \rightarrow$ for $Cl^- + CH_3Cl$ from the TS. The energy profile is compared to the values obtained with an IRC calculation at the MP2/ma-def2-TZVPP level of theory from a MP2 optimised TS. CCSD(T)/ma-def2-TZVPP energy calculations on DFT configurations used numerical gradients over 55 configurations. (b/c) Error in dynamics calculated from 300 K MD simulations propagated with the trained GAP from the MP2 transition state. SOAP only descriptors on C and Cl with $r_c^{SOAP} = 3.5$ Å.

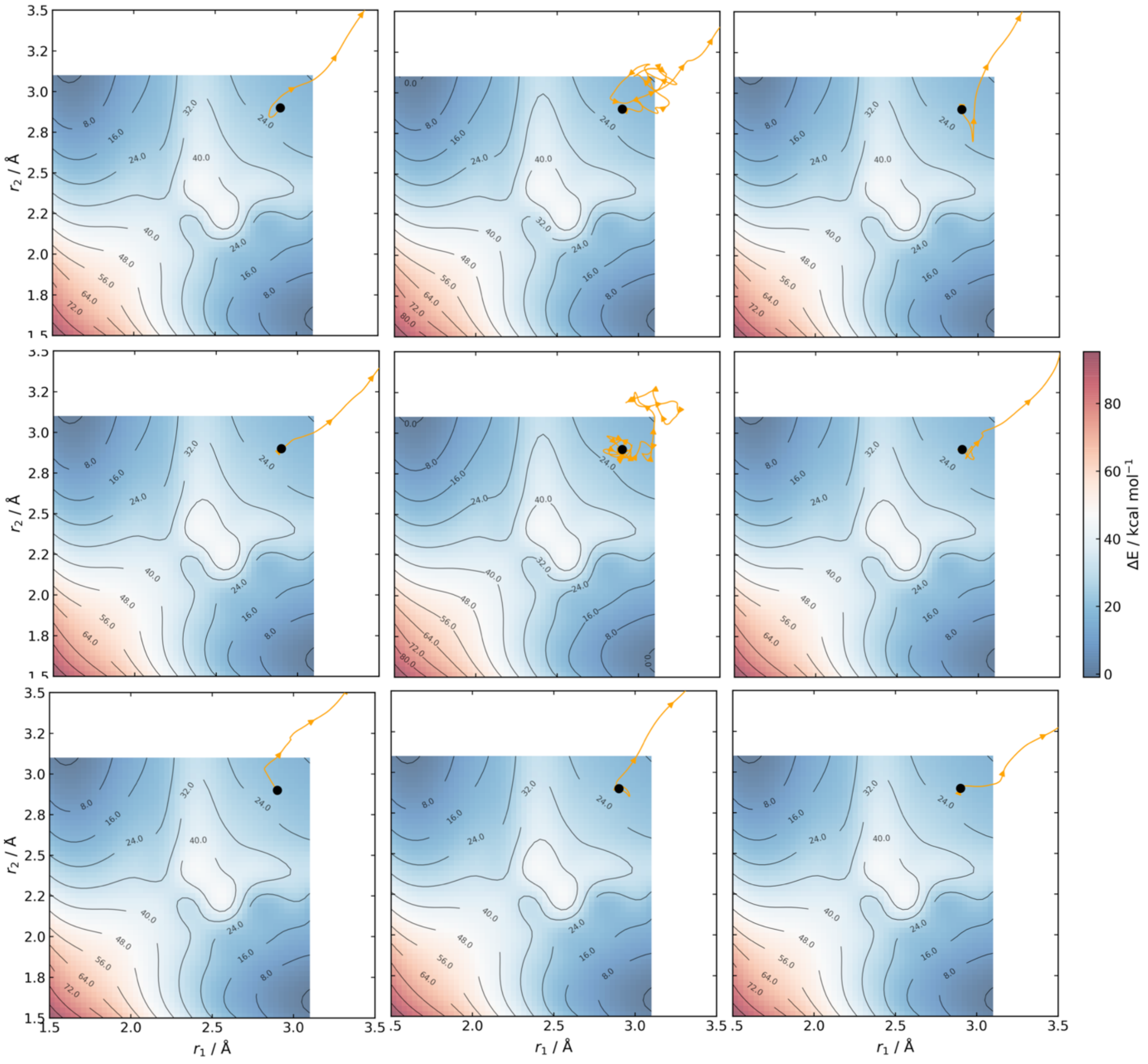

Figure S18. 2D PES along the forming bond distances (r_1, r_2) in the [4+2] dimerization of cyclopentadiene as a function of time. GAP–propagated reactive dynamics (300 K, yellow lines) overlaid on the relaxed 2D PES. **TS**$_1$ (7N in ref. 5) is shown as the black point. All trajectories lead to reactants (cyclopentadiene x 2) after ~100 fs. GAP trained on ground truth B3LYP/def2-SVP. Active learning performed at 500 K initiated at r_1, r_2 = 2.9 Å.

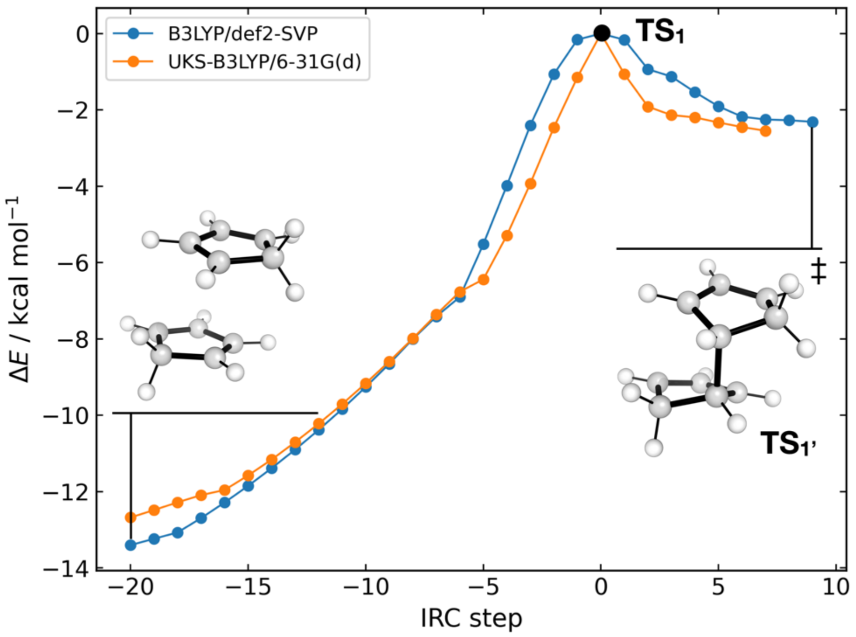

Figure S19. Intrinsic reaction coordinate generated in ORCA from the TS reported in ref. 5 (ν_{imag} = 384.64i cm^{-1}) for the [4+2] dimerization of cyclopentadiene at B3LYP/def2-SVP and unrestricted B3LYP/6-31G(d). Hessian calculations on the forward geometry (**TS**$_{1'}$, ν_{imag} = 152.78i cm^{-1}) indicate a TS to forming products.

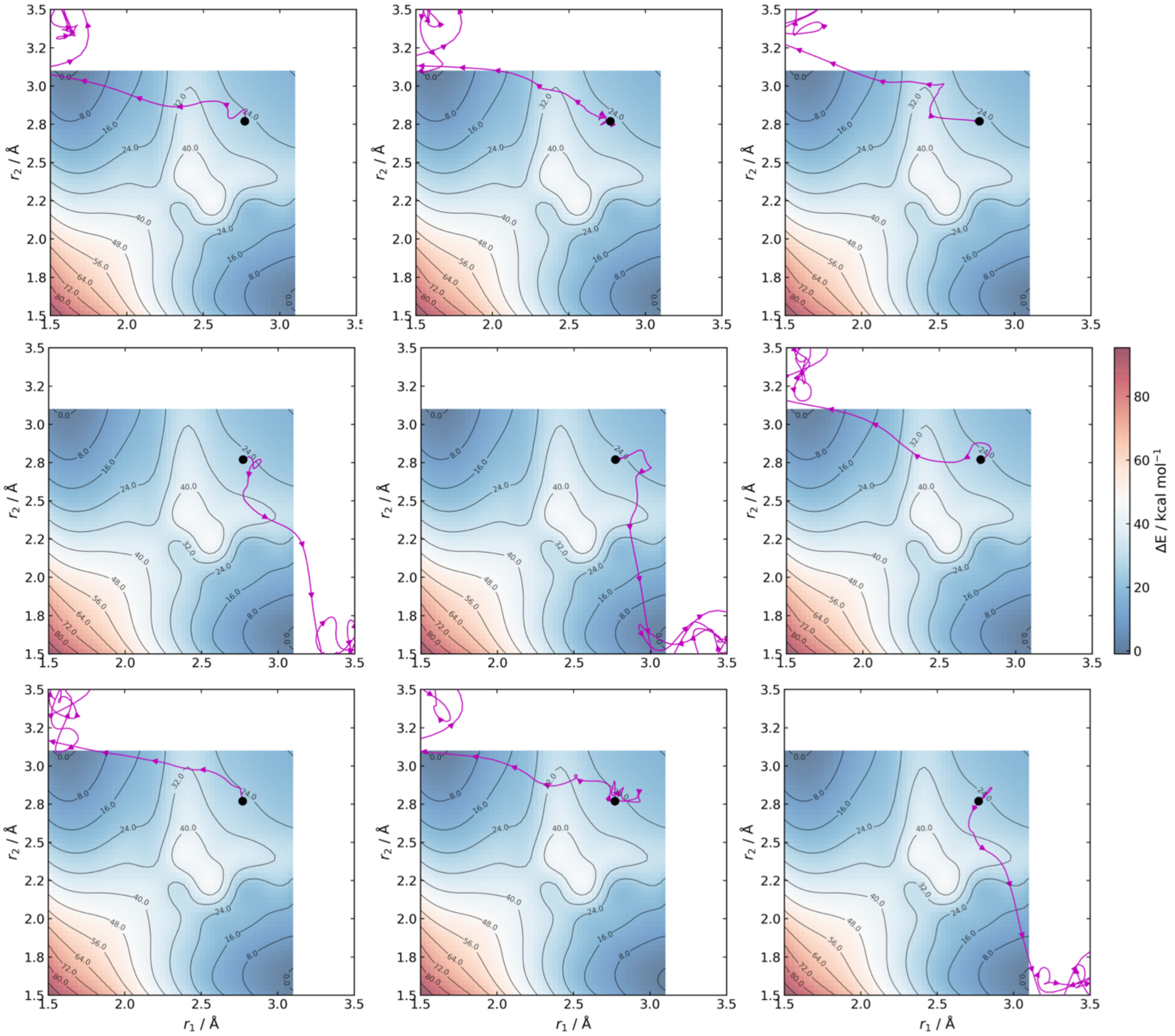

Figure S20. As **Figure S18** but GAP active learning then dynamics initiated from $\mathbf{TS}_{1'}$ (r_1, r_2 = 2.77 Å).

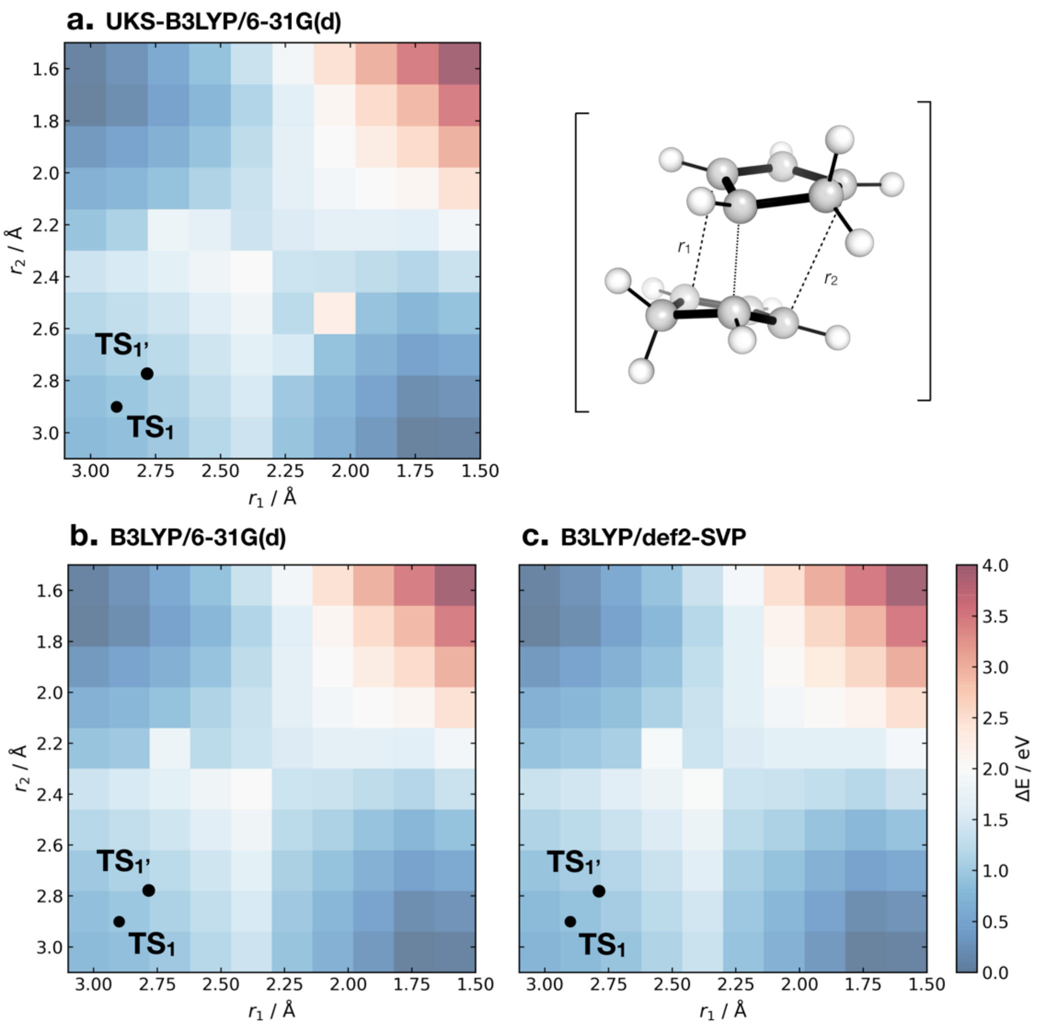

Figure S21. Comparison of 2D relaxed potential energy surfaces at (a) B3LYP/6-31G(d) as reported in ref. 5 and (b) a slightly improved level, B3LYP/def2-SVP.

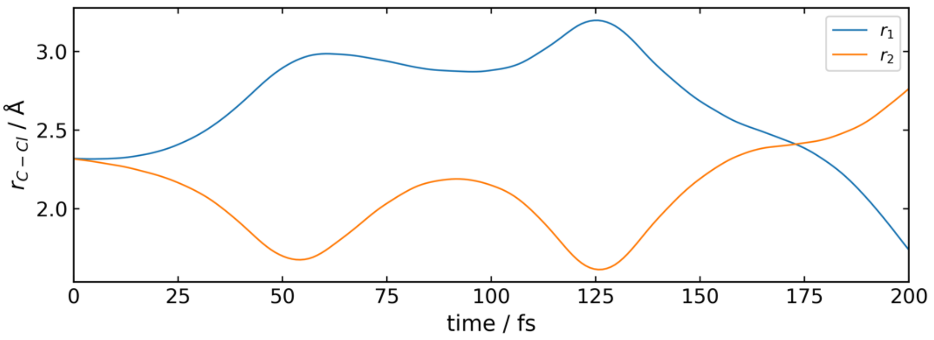

Figure S22. C-Cl distances as a function of time for one of the ten GAP-MD propagated from the TS of Cl+CH_3Cl in explicit water. A barrier recrossing event is observed at ~170 fs.

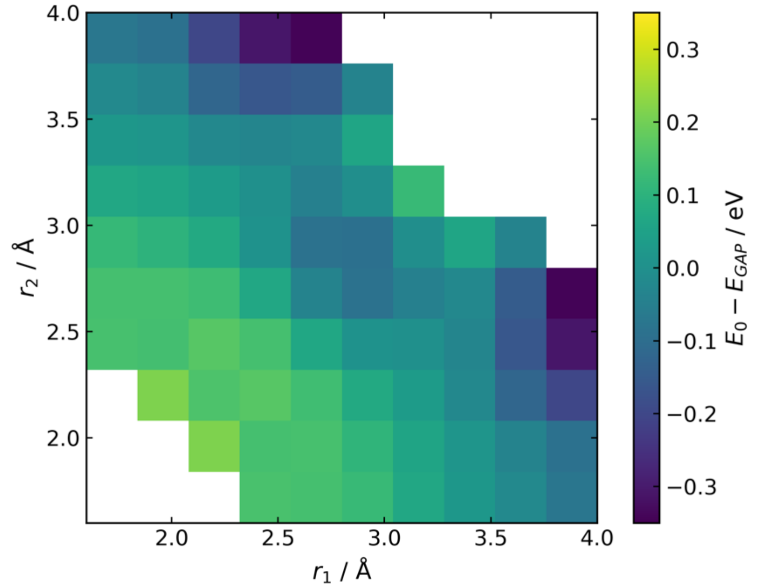

Figure S23. Error between GAP predicted and ground truth (CPCM(Water)-PBE/def2-SVP) referenced to the closest point on the surface to the TS (r_1=r_2=2.4 Å) i.e. the starting point for the GAP active learning at 1600 K. Regions above 2 eV on the ground truth surface masked, as the training explicitly does not include any configurations > 2 eV from the minimum.

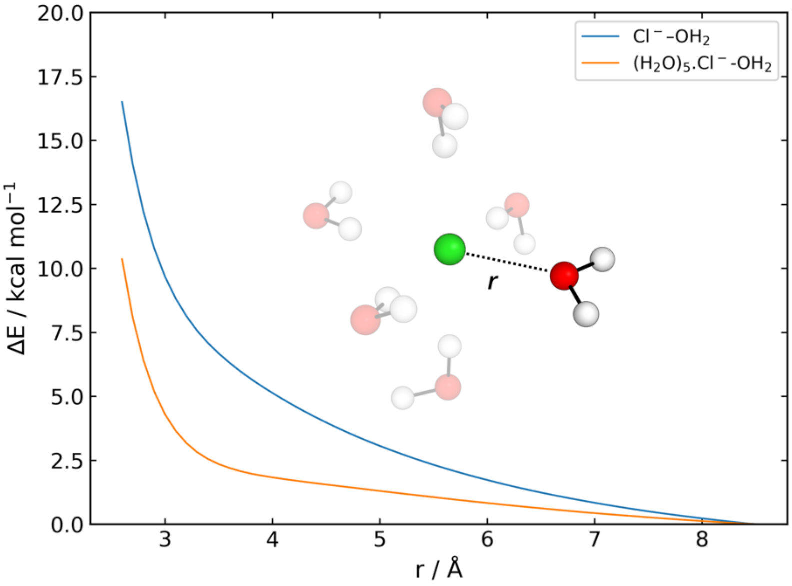

Figure S24. Gas-phase potential energy surfaces for Cl^-–H_2O, with and without a first solvation shell at PBE/ma-def2-TZVP. Coordination number of chloride is 6 from ref. 4. Initial partially solvated chloride generated by hand and optimised with ORCA constraining the Cl–O distance and the O–Cl–O angles to generate a close to octahedral geometry. The solvated ion shows a faster decaying potential; based on this model and r_c^{SOAP} = 4.5 Å was chosen as a compromise between efficiency and accuracy.

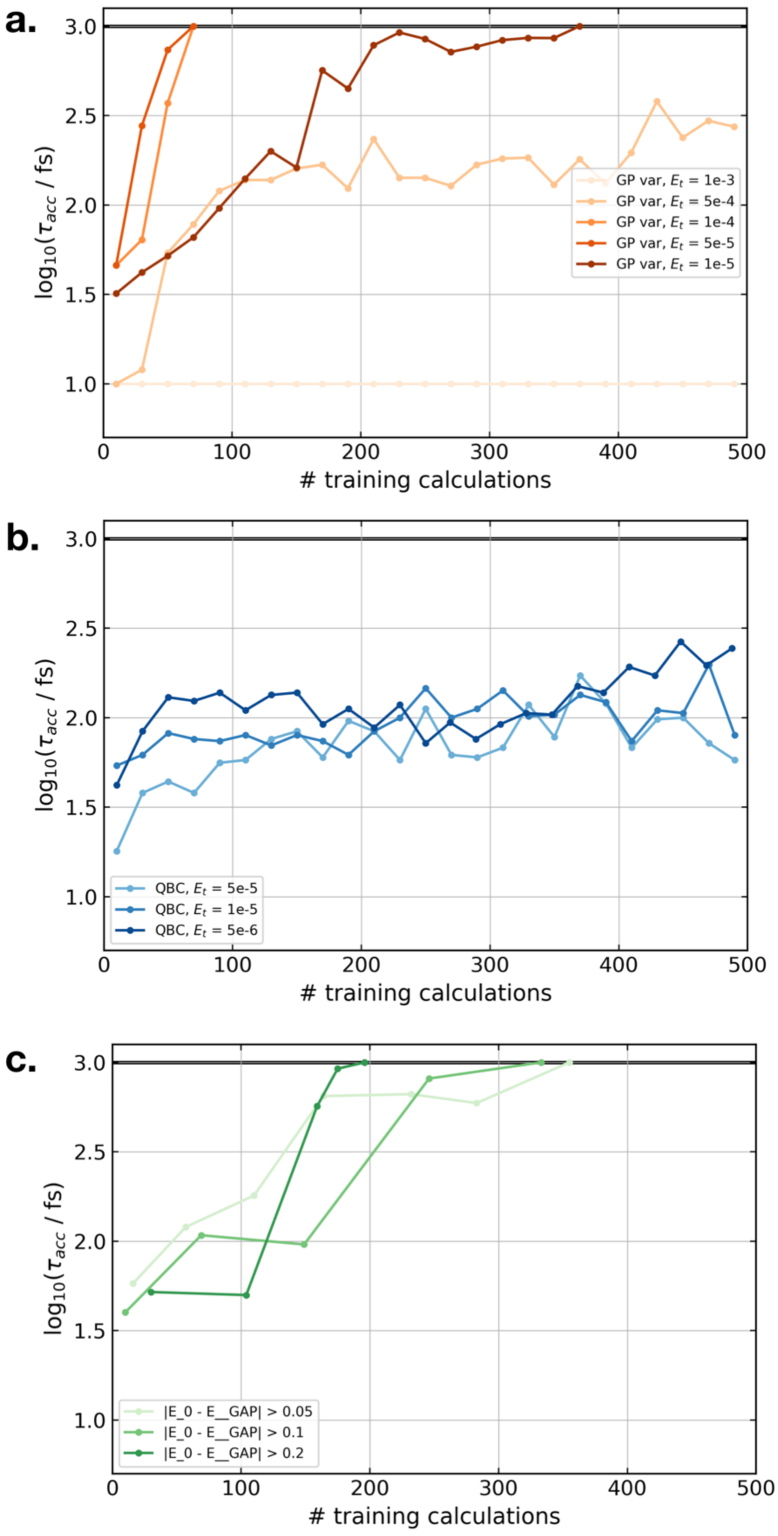

Figure S25. Comparison of selection strategies used in the 'active' learning loop for the intermolecular component of a water GAP trained in a 7 Å cubic box with 10 waters at the DFTB(3ob) level of theory. GAP-MD performed at 300 K with a 0.5 fs timestep for n^3+2 fs iterations sequentially until 1 ps of dynamics was performed. τ_{acc} used a 0.1 eV lower energy threshold and 1 eV total averaged over 5 random initial configurations (identical for each learning curve). (a) Adds a configuration when the maximum atomic energy variance predicted by the Gaussian Process exceeds a threshold E_t (in eV, where the max is taken over all the atoms in a frame of GAP-MD). (b) Query-by-committee (QBC), where a configuration is added if the standard deviation between GAPs trained on the same data (with different random noise) exceeds a threshold E_t (in eV). Values are chosen to span where the first frame from the iterative GAP-MD is chosen to the maximum 1ps allowed. (c) Adds a configuration where the true difference of the total energy exceeds a threshold (in eV).

References

(1) Himanen, L.; Jäger, M. O. J.; Morooka, E. V.; Federici Canova, F.; Ranawat, Y. S.; Gao, D. Z.; Rinke, P.; Foster, A. S. DScribe: Library of Descriptors for Machine Learning in Materials Science. *Comput. Phys. Commun.* **2020**, *247*, 106949.

(2) Zheng, L.; Chen, M.; Sun, Z.; Ko, H.-Y.; Santra, B.; Dhuvad, P.; Wu, X. Structural, Electronic, and Dynamical Properties of Liquid Water by Ab Initio Molecular Dynamics Based on SCAN Functional within the Canonical Ensemble. *J. Chem. Phys.* **2018**, *148* (16), 164505.

(3) Darwent, B. deB. *Bond Dissociation Energies in Simple Molecules: NSRDS-NBS 31*; U.S. National Bureau of Standards.: Washington DC, 1970.

(4) Busch, S.; Pardo, L. C.; O'Dell, W. B.; Bruce, C. D.; Lorenz, C. D.; McLain, S. E. On the Structure of Water and Chloride Ion Interactions with a Peptide Backbone in Solution. *Phys. Chem. Chem. Phys.* **2013**, *15* (48), 21023.

(5) Caramella, P.; Quadrelli, P.; Toma, L. An Unexpected Bispericyclic Transition Structure Leading to 4+2 and 2+4 Cycloadducts in the E Ndo Dimerization of Cyclopentadiene. *J. Am. Chem. Soc.* **2002**, *124* (7), 1130–1131.
